# Prions amplify through degradation of the VPS10P sorting receptor sortilin

**DOI:** 10.1371/journal.ppat.1006470

**Published:** 2017-06-30

**Authors:** Keiji Uchiyama, Mitsuru Tomita, Masashi Yano, Junji Chida, Hideyuki Hara, Nandita Rani Das, Anders Nykjaer, Suehiro Sakaguchi

**Affiliations:** 1 Division of Molecular Neurobiology, Institute for Enzyme Research (KOSOKEN), Tokushima University, Tokushima, Japan; 2 Student Laboratory, Faculty of Medicine, Tokushima University, Tokushima, Japan; 3 Department of Biomedicine, Aarhus University, Aarhus, Denmark; University of Edinburgh, UNITED KINGDOM

## Abstract

Prion diseases are a group of fatal neurodegenerative disorders caused by prions, which consist mainly of the abnormally folded isoform of prion protein, PrP^Sc^. A pivotal pathogenic event in prion disease is progressive accumulation of prions, or PrP^Sc^, in brains through constitutive conformational conversion of the cellular prion protein, PrP^C^, into PrP^Sc^. However, the cellular mechanism by which PrP^Sc^ is progressively accumulated in prion-infected neurons remains unknown. Here, we show that PrP^Sc^ is progressively accumulated in prion-infected cells through degradation of the VPS10P sorting receptor sortilin. We first show that sortilin interacts with PrP^C^ and PrP^Sc^ and sorts them to lysosomes for degradation. Consistently, sortilin-knockdown increased PrP^Sc^ accumulation in prion-infected cells. In contrast, overexpression of sortilin reduced PrP^Sc^ accumulation in prion-infected cells. These results indicate that sortilin negatively regulates PrP^Sc^ accumulation in prion-infected cells. The negative role of sortilin in PrP^Sc^ accumulation was further confirmed in sortilin-knockout mice infected with prions. The infected mice had accelerated prion disease with early accumulation of PrP^Sc^ in their brains. Interestingly, sortilin was reduced in prion-infected cells and mouse brains. Treatment of prion-infected cells with lysosomal inhibitors, but not proteasomal inhibitors, increased the levels of sortilin. Moreover, sortilin was reduced following PrP^Sc^ becoming detectable in cells after infection with prions. These results indicate that PrP^Sc^ accumulation stimulates sortilin degradation in lysosomes. Taken together, these results show that PrP^Sc^ accumulation of itself could impair the sortilin-mediated sorting of PrP^C^ and PrP^Sc^ to lysosomes for degradation by stimulating lysosomal degradation of sortilin, eventually leading to progressive accumulation of PrP^Sc^ in prion-infected cells.

## Author summary

Once prions consisting mainly of PrP^Sc^ infect hosts, they constitutively propagate in their brains. Progressive production of PrP^Sc^ through the constitutive conformational conversion of PrP^C^ into PrP^Sc^ underlies prion propagation. However, the mechanism enabling progressive production of PrP^Sc^ in prion-infected cells remains unknown. We here found that the VPS10P sorting receptor sortilin is involved in degradation of PrP^C^ and PrP^Sc^ in infected cells by binding to both molecules and subsequently trafficking them to the lysosomal protein degradation pathway. Interestingly, we also found that degradation of sortilin was stimulated in lysosomes in prion-infected cells possibly as a result of the sortilin-PrP^C^ or -PrP^Sc^ complexes being trafficked to lysosomes. Our findings indicate that PrP^Sc^ itself impairs the sortilin-mediated degradation of PrP^C^ and PrP^Sc^ by stimulating degradation of sortilin in lysosomes. This eventually results in progressive production of PrP^Sc^ in prion-infected cells by increasing the opportunity of PrP^C^ to convert into PrP^Sc^ and by accumulating the already produced PrP^Sc^. This mechanism was confirmed in sortilin-KO mice infected with prions. The mice had exacerbated prion disease with earlier accumulation of PrP^Sc^ in their brains.

## Introduction

Prion diseases are a group of fatal neurodegenerative disorders, which include Creutzfeldt-Jakob disease in humans and bovine spongiform encephalopathy and scrapie in animals [1]. They are caused by the infectious agents termed prions, which mainly consist of the abnormally folded, amyloidogenic isoform of prion protein, designated PrP^Sc^. PrP^Sc^ is a β-sheet-rich conformer produced by conformational conversion of the cellular counterpart, PrP^C^ [1]. Intermolecular interaction between PrP^C^ and PrP^Sc^ is essential for the conversion of PrP^C^ into PrP^Sc^. We and others have shown that mice devoid of PrP^C^ neither developed the disease nor accumulated PrP^Sc^ even after prions were inoculated into their brains [2–5]. These results indicate that the conversion of PrP^C^ into PrP^Sc^ plays a pivotal role in the pathogenesis of prion disease, and that depletion of PrP^C^ could be therapeutic by preventing the production of PrP^Sc^.

PrP^C^ is normally located at the cell surface as a glycosylphosphatidylinositol (GPI)-anchored glycoprotein [6]. Some endocytosed PrP^C^ molecules are transported to lysosomes for degradation while others are recycled to the cell surface through the endocytic recycling compartments [7]. PrP^Sc^ is also trafficked to lysosomes for degradation [7]. However, the cellular transport mechanism of PrP^C^ and PrP^Sc^ to lysosomes remains unknown. Whether prion infection or PrP^Sc^ impairs the lysosomal trafficking of PrP^C^ and PrP^Sc^ for its progressive propagation is also unknown.

The vacuolar protein sorting-10 protein (VPS10P)-domain receptors, including sortilin, SorLA, SorCS1, SorCS2 and SorCS3, are multi-ligand type I transmembrane proteins abundantly expressed in the brain and involved in neuronal function and viability [8,9]. They function as a cargo receptor to deliver a number of cargo proteins to their subcellular destination through the VPS10P domain in the extracellular luminal N-terminus. Sortilin traffics the amyloid precursor protein (APP)-cleaving enzyme BACE1 [10] and the neurotrophic factor receptors Trks [11]. SorLA directs trafficking of APP into the recycling pathway [12]. SorCS1 also mediates APP transport [13]. Recent lines of evidence indicate that the altered VPS10P receptor-mediated trafficking could be involved in the pathogenesis of neurodegenerative disorders, including Alzheimer’s disease (AD) [12–15] and frontotemporal lobar degeneration (FTLD) [16]. However, the role of VPS10P receptors in the trafficking of PrP^C^ or PrP^Sc^ and in the pathogenesis of prion disease is little known.

In the present study, we show that sortilin has an inhibitory role in PrP^Sc^ accumulation by sorting PrP^C^ and PrP^Sc^ to lysosomes for degradation. Interestingly, however, prion infection stimulates lysosomal degradation of sortilin, indicating that prion infection itself could disturb the inhibitory function of sortilin. We also confirm that sortilin-knockout (KO) mice have accelerated prion disease after infection with RML prions, with early accumulation of PrP^Sc^ in their brains. These results suggest that PrP^Sc^ accumulation may be amplified through PrP^Sc^-induced impairment of the sortilin-mediated lysosomal degradation of PrP^C^ and PrP^Sc^.

## Results

### Sortilin is a novel PrP^C^-binding protein regulating the surface levels of PrP^C^

**Fig 1:**
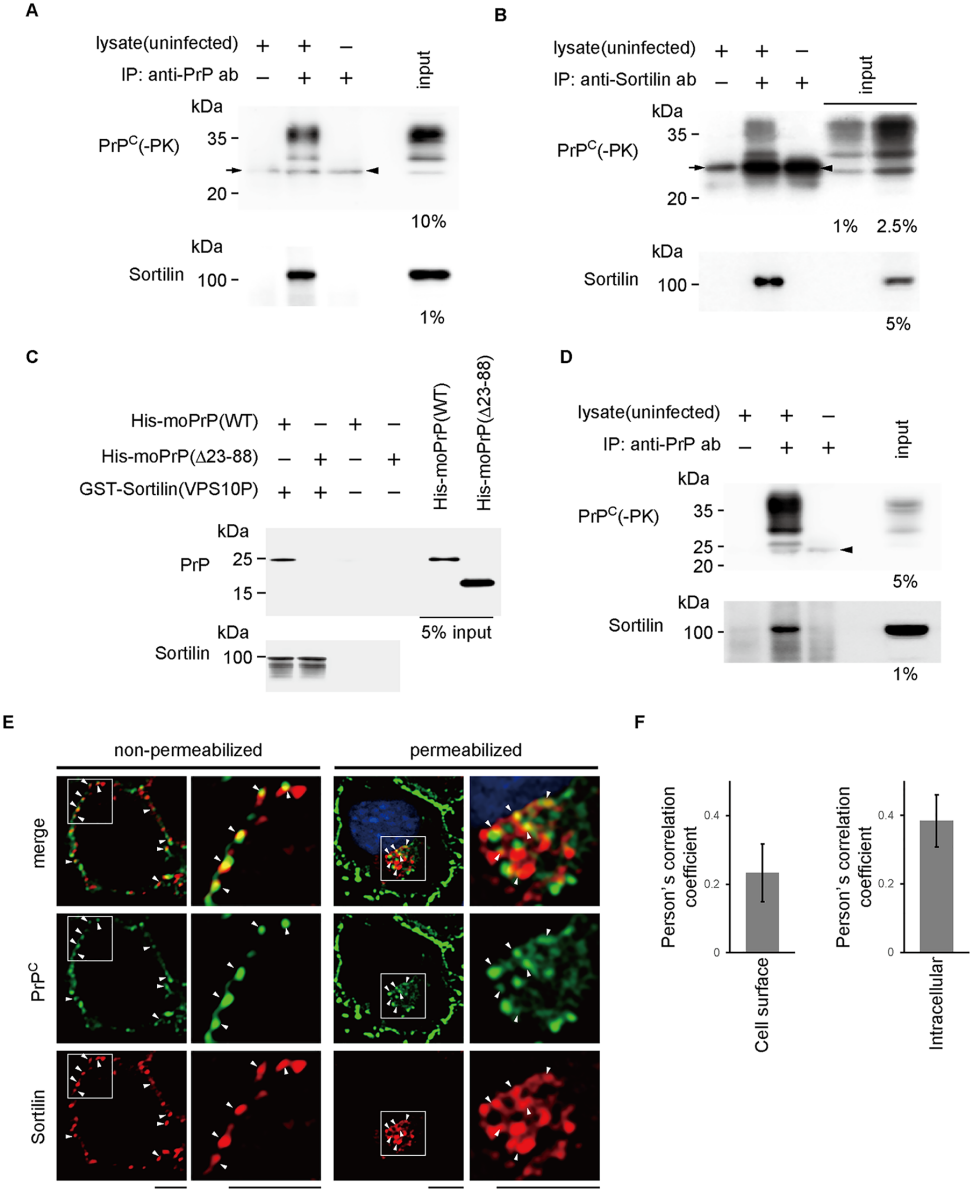
PrP^C^ directly interacts with sortilin on the cell surface and inside cells. (A, B) Co-immunoprecipitation assay in N2aC24 cells with SAF61 anti-PrP Ab (A) or rabbit polyclonal anti-sortilin Abs (B). Arrows and arrowheads indicate a non-specific signal of the degraded fragment of protein G or the light chain of antibodies used in co-immunoprecipitation. Percentages indicate the proportion of the sample loaded. (C) GST-pulldown assay using GST-tagged VPS10P domain of sortilin and His-tagged full-length recombinant PrP or His-tagged recombinant PrP lacking residues 23–88. Percentages indicate the proportion of the sample loaded. (D) Co-immunoprecipitation assay in mouse brain homogenate with SAF61 anti-PrP Ab. Percentages indicate the proportion of the sample loaded. Arrowheads indicate a non-specific signal of the degraded fragment of protein G. (E) Double immunofluorescence staining of PrP^C^ (green) and sortilin (red) in permeabilized or non-permeabilized N2aC24 cells, with SAF83 anti-PrP Ab and goat polyclonal anti-sortilin Abs. Arrowheads indicate co-localized signals of PrP^C^ and sortilin. Bar, 5 μm. (F) Person’s correlation coefficient for cell surface and intracellular co-localization of PrP^C^ and sortilin.

To investigate the role of VPS10P cargo receptors in the trafficking of PrP^C^, we first examined whether or not VPS10P molecules could interact with PrP^C^. Co-immunoprecipitation assay in neuroblastoma N2aC24 cells showed that SAF61 anti-PrP antibody (Ab) precipitated PrP^C^ with sortilin, but not with other VPS10P molecules (Fig 1A, S1 Fig). PrP^C^ was also co-precipitated with sortilin by anti-sortilin Abs (Fig 1B). GST-pulldown assay using purified recombinant proteins revealed that the VPS10P domain of sortilin fused with GST (GST-VPS10P) successfully pulled down His-tagged full-length recombinant PrP, but not PrP with a deletion of 23–88 residues (Fig 1C), suggesting that the residues 23–88 are important for PrP^C^ to interact with sortilin. SAF61 anti-PrP Ab also co-precipitated full-length mycHis-tagged sortilin expressed in sortilin-KO N2aC24 cells, designated ΔSort#1 cells, but not in PrP-KO N2a cells, N2aΔPrP#1 cells (S2A and S2B Fig). Both types of KO cells were established using the CRISPR-Cas genome editing system. This clearly indicates that PrP^C^ expression is required for sortilin to be co-precipitated by SAF61 anti-PrP Ab, further supporting the interaction of sortilin and PrP^C^. However, sortilin lacking residues 610–753 was not efficiently co-precipitated with the Ab, compared to other deletion mutants of sortilin (S2A and S2B Fig), suggesting that the residues 610–753 of sortilin are involved in interaction with PrP^C^. Furthermore, co-immunoprecipitation assay using mouse brain homogenates also revealed an interaction between PrP^C^ and sortilin (Fig 1D). Immunofluorescence staining of non-permeabilized N2aC24 cells showed co-localization of sortilin and PrP^C^ on the cell surface (Fig 1E and 1F; S3 Fig). Intracellular co-localization of sortilin and PrP^C^ was also observed in permeabilized cells (Fig 1E and 1F; S3 Fig).

To further investigate PrP^C^ interaction with sortilin on the cell surface, we labeled PrP^C^ on the cell surface of N2aC24 cells with SAF61 anti-PrP Ab, lysed the cells, and incubated the lysate with protein-G-conjugated Dynabeads (S4A Fig). The Ab-labeled PrP^C^ complexes were collected using magnet (S4A Fig). N2aΔPrP#1 cells were used as negative control (S4A Fig). Sortilin was co-collected with PrP^C^ from N2aC24 cells, but not from N2aΔPrP#1 cells (S4B Fig). Sortilin was similarly expressed in N2aC24 and N2aΔPrP#1 cells (S4C Fig). These results further support PrP^C^ interacting with sortilin on the cell surface.

**Fig 2:**
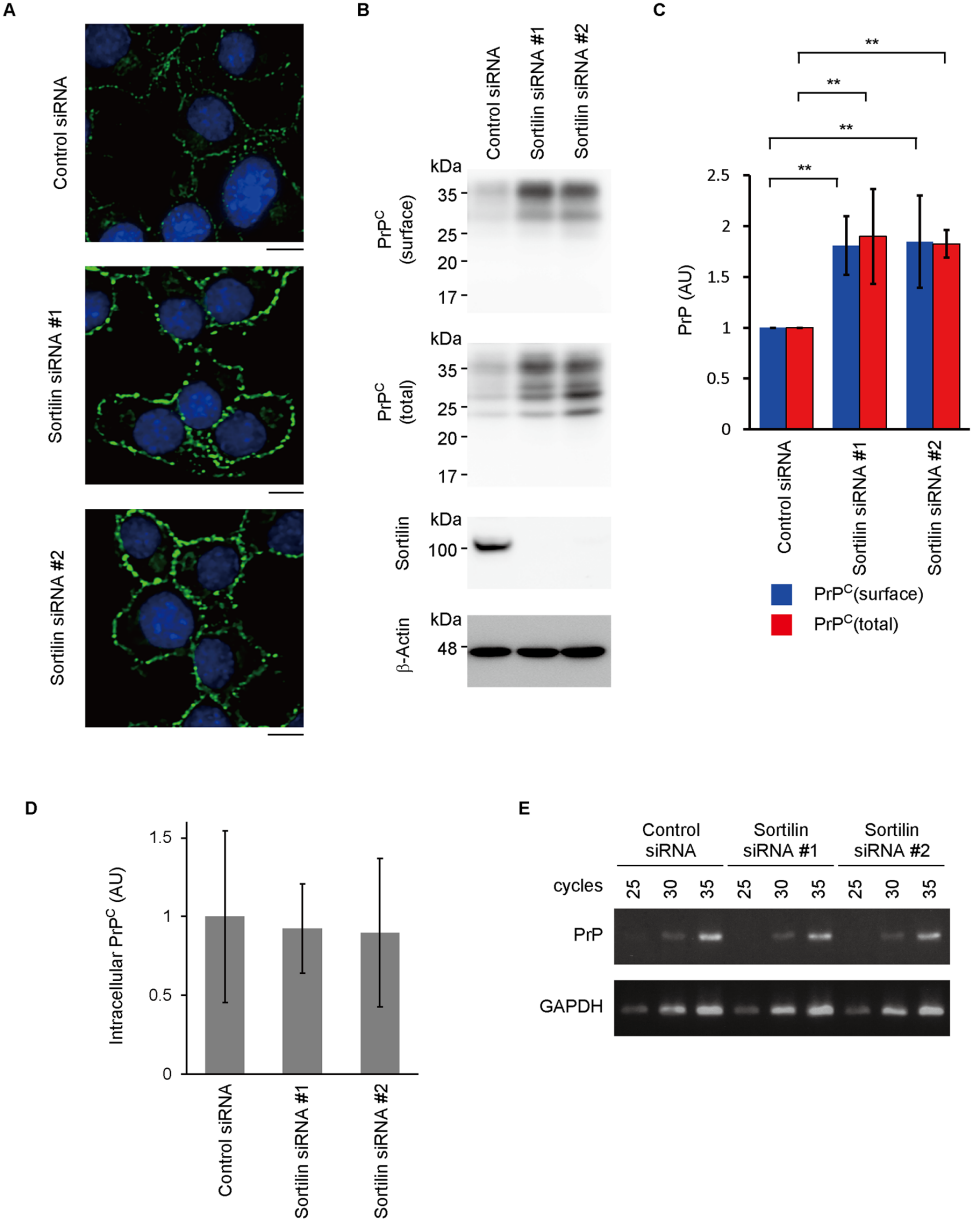
Knockdown of sortilin increases PrP^C^ on the cell surface. (A) Immunofluorescence staining of PrP^C^ with SAF83 anti-PrP Ab in permeabilized N2aC24 cells 6 days after transfection with control or sortilin-specific siRNAs. Bar, 10 μm. (B) Biotin labeling of surface proteins in N2aC24 cells 6 days after transfection with control or sortilin-specific siRNAs. The biotinylated surface proteins were purified using NeutrAvidin beads. The surface (biotinylated) PrP^C^ and total (biotinylated+unbiotinylated) PrP^C^ were analyzed by Western blotting with 6D11 anti-PrP Ab. (C) Quantification of PrP^C^ in (B) after normalization against β-actin. Each signal intensity in Sort-KD cells was evaluated against that in control cells. Data are means ± standard deviation (SD) of 3 independent experiments. ** p < 0.01. (D) Quantification of intracellular PrP^C^ in (A). Data are means ± SD of 9 cells for each cell type. (E) RT-PCR for PrP in N2aC24 cells transfected with control siRNA or sortilin-specific siRNAs. GAPDH, glyceraldehyde-3-phosphate dehydrogenase.

We then knocked down sortilin in N2aC24 cells using two sortilin-specific siRNAs, termed siRNA#1 and #2. Immunostaining of sortilin-knockdown (Sort-KD) cells showed an increase in PrP^C^ expression on the cell surface (Fig 2A). Biotin labeling of surface proteins confirmed the increased surface levels of PrP^C^ in Sort-KD cells (Fig 2B and 2C). Total PrP^C^ levels were also increased in Sort-KD cells (Fig 2B and 2C). However, intracellular PrP^C^ was not increased in Sort-KD cells (Fig 2D), indicating that the surface PrP^C^ is specifically increased in Sort-KD cells. PrP mRNA levels were not increased in Sort-KD cells (Fig 2E), suggesting that the increased surface expression of PrP^C^ might be attributable to the impaired degradation of PrP^C^ in Sort-KD cells. PrP^C^ levels were also significantly increased in the brains of sortilin-KO (Sort1^-/-^) mice compared to those in wild-type (WT) mice (S5A and S5B Fig).

PrP^C^ undergoes an endopeptidic cleavage by the ADAM family of metalloproteases, with the C-terminal fragment, designated the C1 fragment, being produced [17,18]. Sort-KD cells produced the C1 fragment more abundantly than N2aC24 cells (S6A Fig). This is probably because PrP^C^ was increased on the cell surface in Sort-KD cells. We also investigated PrP^C^ levels in exosomes of N2aC24 and sortilin-KO ΔSort#1 and ΔSort#2 cells. ΔSort#1 and ΔSort#2 cells also showed an increase in total PrP^C^ levels (S6B Fig). PrP^C^ was significantly higher in exosomes from ΔSort#1 and #2 cells than in those from N2aC24 cells (S6B and S6C Fig). Exosomes were verified by the presence of exosome-specific molecules TSG101 and flotillin and the absence of GM130 and Bcl-2, both of which are not normally included in exosomes (S6B Fig) [19,20].

### Sortilin sorts surface PrP^C^ to late endosomes/lysosomes

**Fig 3:**
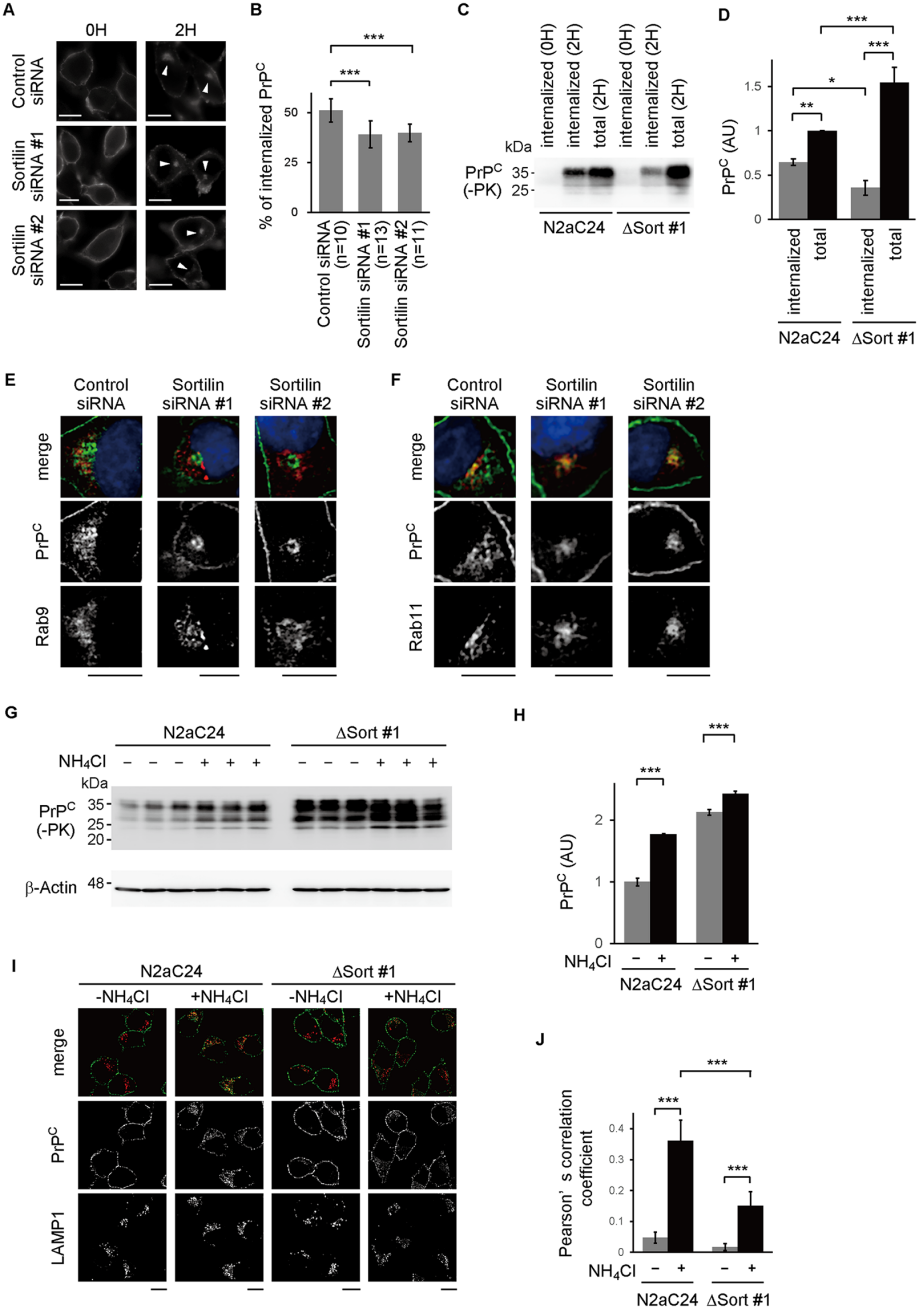
Sortilin is an endocytic receptor for PrP^C^ to late endosomes. (A) Representative images of the SAF61 Ab-labeled PrP^C^ allowed to be endocyosed in N2aC24 cells after transfection with control or sortilin-specific siRNAs. The labeled PrP^C^ was visualized with Alexa Fluoro 488 goat anti-mouse IgG Abs. Arrowheads indicate signals of the internalized PrP^C^. Bar, 10 μm. (B) Quantification of the labeled PrP^C^ internalized against the total labeled PrP^C^ in (A). Data are means ± SD of 10–13 cells. *** p < 0.001. (C) Internalization assay of biotinylated cell surface PrP^C^ in N2aC24 cells and sortilin-KO N2aC24 cells, or ΔSort#1 cells. The cell surface proteins were biotinylated and allowed to internalize for 2 h. Biotins were removed only from cell surface proteins but not from those already internalized using reducing glutathione. The biotin-labeled internalized proteins were purified using avidin-beads and subjected to Western blotting with 6D11 anti-PrP Ab. (D) Signal intensities in each lane in Sort-KD cells were evaluated against that of total PrP signals in N2aC24 cells. Data are means ± SD of 3 independent experiments. * p < 0.05, ** p < 0.01, *** p < 0.001. (E, F) Double immunofluorescence staining of the internalized, SAF61-labeled PrP^C^ (green) and the late endosome marker Rab9 (red) or the recycling endosome marker Rab11 (red) in permeabilized N2aC24 cells transfected with control or sortilin-specific siRNAs. Bar, 10 μm. (G) Western blotting for PrP^C^ and sortilin in N2aC24 and sortilin-KO (ΔSort#1) cells after 12 h-treatment with or without 20 mM NH_4_Cl. (H) Quantification of PrP^C^ in (G) after normalization against β-actin. Signal intensity in NH_4_Cl-treated cells was evaluated against that in NH_4_Cl-untreated N2aC24 cells. Data are means ± SD of 3 independent experiments. *** p < 0.001. (I) Double immunofluorescence staining of PrP^C^ and the lysosome marker LAMP1 in N2aC24 and sortilin-KO (ΔSort#1) cells after 12 h-treatment with 20 mM NH_4_Cl. Bar, 10 μm. (J) Pearson’s correlation coefficient for co-localization of PrP^C^ and LAMP1 in N2aC24 cells treated (n = 106) and untreated (n = 151) with NH_4_Cl and in ΔSort#1 cells treated (n = 140) and untreated (n = 166) with NH_4_Cl. Data are means ± SD. *** p < 0.001.

To address whether or not sortilin could sort surface PrP^C^ to lysosomes for degradation, we first investigated the role of sortilin in internalization of surface PrP^C^ using an Ab-labeling technique. Surface PrP^C^ was labeled with SAF61 anti-PrP Ab at 4°C in which internalization of membrane proteins is inhibited, and then allowed to be internalized for 2 h at 37°C. The labeled PrP^C^ was then detected using Alexa Fluoro 488-conjugated anti-mouse IgG Abs. The internalization of the labeled PrP^C^ was slightly but significantly inhibited in Sort-KD cells, compared to that in control N2aC24 cells (Fig 3A and 3B), suggesting that sortilin could be involved in internalization of some portions of PrP^C^. To further confirm the involvement of sortilin in internalization of PrP^C^, we biotinylated the surface proteins of N2aC24 cells and sortilin-KO (ΔSort) cells #1 with sulfo-NHS-SS-biotin, whose biotin motif can be removed by reducing agents. We then allowed the biotinylated proteins to be internalized for 2 h, and treated the cells with the membrane-impermeable reducing agent glutathione to remove the biotins only from surface proteins but not from those internalized. The treated cells were lysed, and then biotin-labeled, internalized proteins were purified using avidin-beads, and investigated for internalized PrP^C^ by Western blotting with 6D11 anti-PrP Ab. Strong signals corresponding to the internalized PrP^C^ were detected in N2aC24 cells (Fig 3C and 3D). However, the signals were significantly reduced in ΔSort#1 cells (Fig 3C and 3D). These results reinforce the role of sortilin in internalization of PrP^C^.

To track the SAF61 anti-PrP Ab-labeled, internalized PrP^C^ in N2aC24 and Sort-KD cells, we immunofluorescently stained both types of cells for internalized PrP^C^ with the late endosome marker Rab9 or the recycling endosome marker Rab11. The labeled PrP^C^ was normally internalized to both late endosomes and recycling endosomes, as observed in N2aC24 cells (Fig 3E and 3F; S7A–S7D Fig). However, in Sort-KD cells, localization of PrP^C^ was markedly shifted from the late endosomes (Fig 3E; S7A and S7B Fig) to the recycling endosomes (Fig 3F; S7C and S7D Fig). These results indicate that internalization of PrP^C^ to the recycling endosomes could be independent of sortilin. Furthermore, sortilin could function to sort surface PrP^C^ to the late endosome/lysosome degradation pathway, thereby regulating levels of surface PrP^C^. Consistent with the results from Sort-KD cells, sortilin-KO ΔSort#1 cells showed higher expression of PrP^C^ than control N2aC24 cells (Fig 3G and 3H). Inhibition of lysosomal enzymes by NH_4_Cl increased PrP^C^ markedly in N2aC24 cells, but only slightly in ΔSort#1 cells (Fig 3G and 3H). PrP^C^ was detected in the LAMP1-positive lysosomes in both cell types after NH_4_Cl treatment (Fig 3I and 3J). However, its lysosomal localization was much less in ΔSort#1 cells than in N2aC24 cells (Fig 3I and 3J). These results confirm that transport of PrP^C^ to lysosomes is disturbed in sortilin-deficient cells, therefore reducing the localization of PrP^C^ in lysosomes and resulting in an increase in PrP^C^ levels in sortilin-deficient cells.

We also investigated localization of PrP^C^ in early endosomes in N2aC24 and Sort-KD cells. PrP^C^ was labeled with SAF61 Ab and spontaneously internalized for 1 h instead of 2 h, which was utilized for detection of internalized PrP^C^ in late endosomes or recycling endosomes, because PrP^C^ may be only transiently located in early endosomes before being trafficked to late endosomes/lysosomes or recycling endosomes. Indeed, only a very small fraction of PrP^C^ was detected in the EAA1-positive early endosomes in N2aC24 cells (S7E and S7F Fig). PrP^C^ was slightly, but not significantly, less localized in early endosomes in Sort-KD cells than in N2aC24 cells (S7E and S7F Fig). This might be consistent with the sortilin-independent trafficking of PrP^C^ being active through early endosomes.

We also investigated the internalization of PrP with a deletion of 23–88 residues, PrPΔ23–88, to lysosomes by establishing PrP-KO N2aΔPrP cells permanently expressing wild-type (WT) mouse PrP^C^ (WT#1 and #2) or PrPΔ23–88 (Δ23–88#1 and #2). Western blotting showed that PrPΔ23–88 was expressed at higher levels than WT PrP^C^ without NH_4_Cl treatment (S8A and S8B Fig), suggesting that PrPΔ23–88 might be less degraded than WT PrP^C^. NH_4_Cl treatment only slightly increased PrPΔ23–88 in Δ23–88#1 and #2 cells, but markedly increased WT PrP^C^ in WT#1 and #2 cells (S8A and S8B Fig). NH_4_Cl treatment also only slightly increased PrPΔ23–88 in the LAMP1-positive lysosomes in Δ23–88#1 and #2 cells, but markedly increased WT PrP^C^ in the lysosomes in WT#1 and #2 cells (S8C and S8D Fig). These results suggest that residues 23–88 could be involved in trafficking of PrP^C^ to lysosomes for degradation. Sortilin expression was unaffected in Δ23–88#1 and #2 cells (S8E and S8F Fig). No interaction between sortilin and PrPΔ23–88 was detected (S8G Fig). It is thus conceivable that the interaction with sortilin could be important for PrP^C^ transportation to lysosomes for degradation. However, the increased localization of PrPΔ23–88 in lysosomes after NH_4_Cl treatment in Δ23–88#1 and #2 cells suggests that other molecules are also involved in trafficking of PrP^C^ to lysosomes.

### PrP^C^ shifts to raft domains in sortilin-KO cells

**Fig 4:**
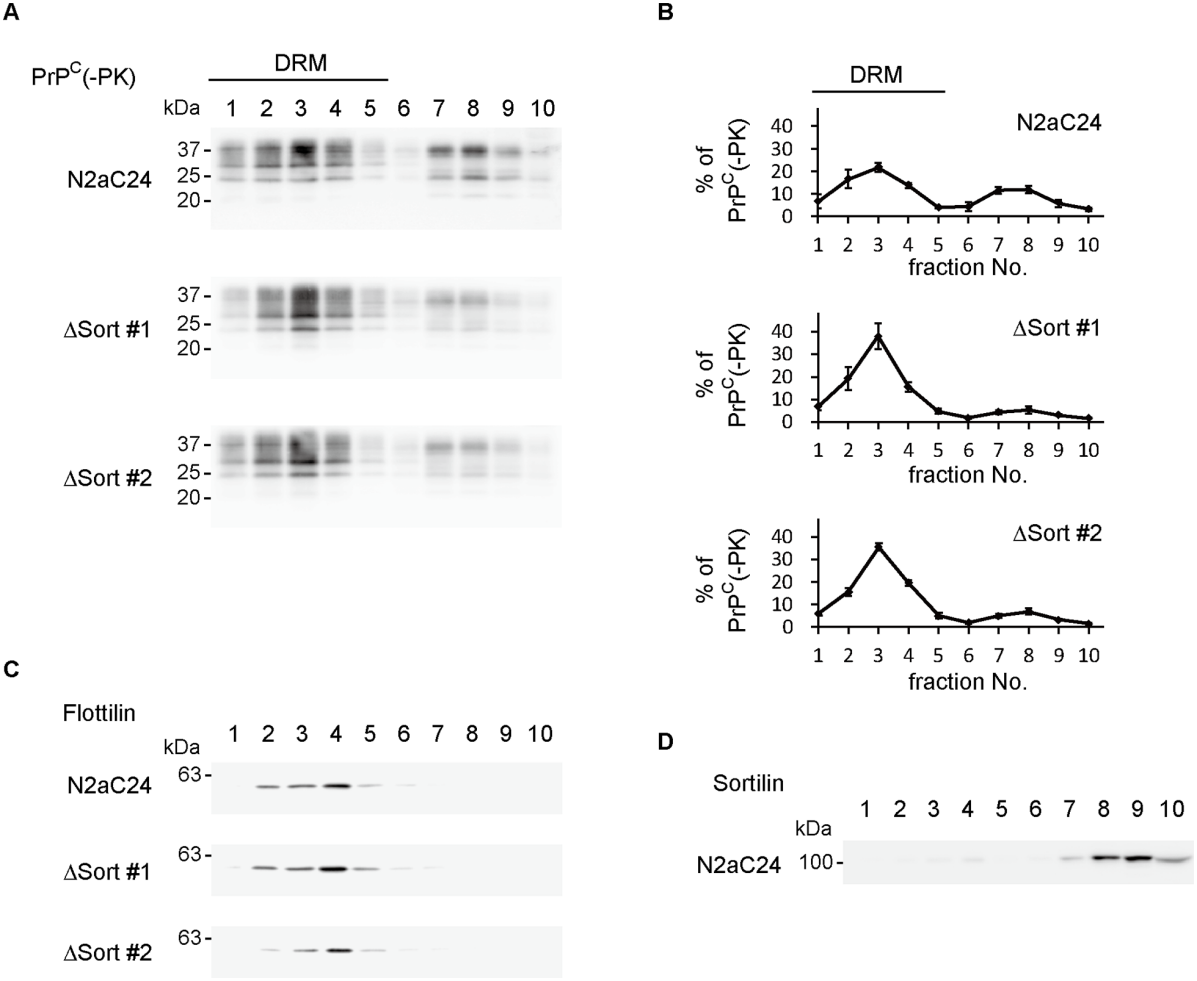
Membrane microdomain distribution of PrP^C^ is changed in sortilin-KO cells. (A) Discontinuous sucrose gradient centrifugation of N2aC24 and sortilin-KO (ΔSort#1 and #2) cells was carried out to see membrane microdomain distribution of PrP^C^. (B) Quantification of PrP^C^ in each fraction against the total PrP^C^ in (A). The signal density in each lane was evaluated against the total signal density of all lanes. Data are means ± SD of 3 independent experiments. (C) Discontinuous sucrose gradient centrifugation of N2aC24 and sortilin-KO (ΔSort#1 and #2) cells was carried out to see membrane microdomain distribution of flotillin-2. (D) Discontinuous sucrose gradient centrifugation of uninfected N2aC24 for sortilin.

To gain insight into the mechanism of sortilin-mediated sorting of PrP^C^ to lysosomes, we investigated membrane microdomain localization of PrP^C^ in N2aC24 and sortilin-KO N2aC24 cells, ΔSort#1 and ΔSort#2 cells, by detergent-based biochemical membrane fractionation. PrP^C^ was detected in both detergent-resistant membrane (DRM) and detergent-soluble membrane fractions, that is raft and non-raft fractions, in N2aC24 cells, with higher amounts of PrP^C^ in raft fractions (63.0%) than in non-raft fractions (37.0%) (Fig 4A and 4B). However, in ΔSort#1 and #2 cells, PrP^C^ in non-raft fractions was reduced to 11.9 and 14.9%, respectively (Fig 4A and 4B). Instead, PrP^C^ was increased in raft fractions (Fig 4A and 4B). These results suggest that sortilin could shift the localization of PrP^C^ to non-raft domains from raft domains. Raft-resident protein flotillin-2 was observed in raft fractions in N2aC24 and ΔSort cells (Fig 4C), ruling out the possibility that lack of sortilin could affect the membrane microdomain integrity leading to the shift in the location of surface PrP^C^ in ΔSort cells. Sortilin was predominantly detected in non-raft fractions in N2aC24 and ΔSort cells (Fig 4D), indicating that sortilin is a non-raft protein. We also performed the membrane fractionation assay for PrP^C^-expressing WT#1 and #2 cells and PrPΔ23-88-expressing Δ23–88#1 and #2 cells. PrP^C^ was detected in raft and non-raft fractions in WT cells (S9A and S9B Fig). However, PrPΔ23–88 increased its localization at raft fractions (S9A and S9B Fig). These results indicate that residues 23–88 are important for PrP^C^ to be retained at non-raft domains. We then investigated membrane microdomain localization of PrP^C^ in the brains of Sort1^-/-^ and WT mice. Localization of PrP^C^ at raft and non-raft fractions was observed in WT brains (S9C and S9D Fig). However, PrP^C^ was increased in raft fractions in Sort1^-/-^ brains (S9C and S9D Fig). Taken together, these results suggest that sortilin might function to retain surface PrP^C^ in non-raft domains and sort the non-raft PrP^C^ to the late endosome/lysosome degradation pathway through interaction with residues 23–88 of PrP^C^.

We also investigated membrane microdomain location of PrP molecules in prion-infected N2aC24L1-3 cells. In contrast to PrP^C^ detected in raft and non-raft domains in uninfected N2aC24 cells (Fig 4A and 4B), total PrP molecules and PK-resistant PrP^Sc^ were predominantly detected in raft fractions in prion-infected N2aC24L1-3 cells (S10A and S10B Fig). These results suggest that prion infection accumulates PrP^Sc^ and PrP^C^ in raft domains.

### Sortilin interacts with PrP^Sc^ and facilitates its degradation

**Fig 5:**
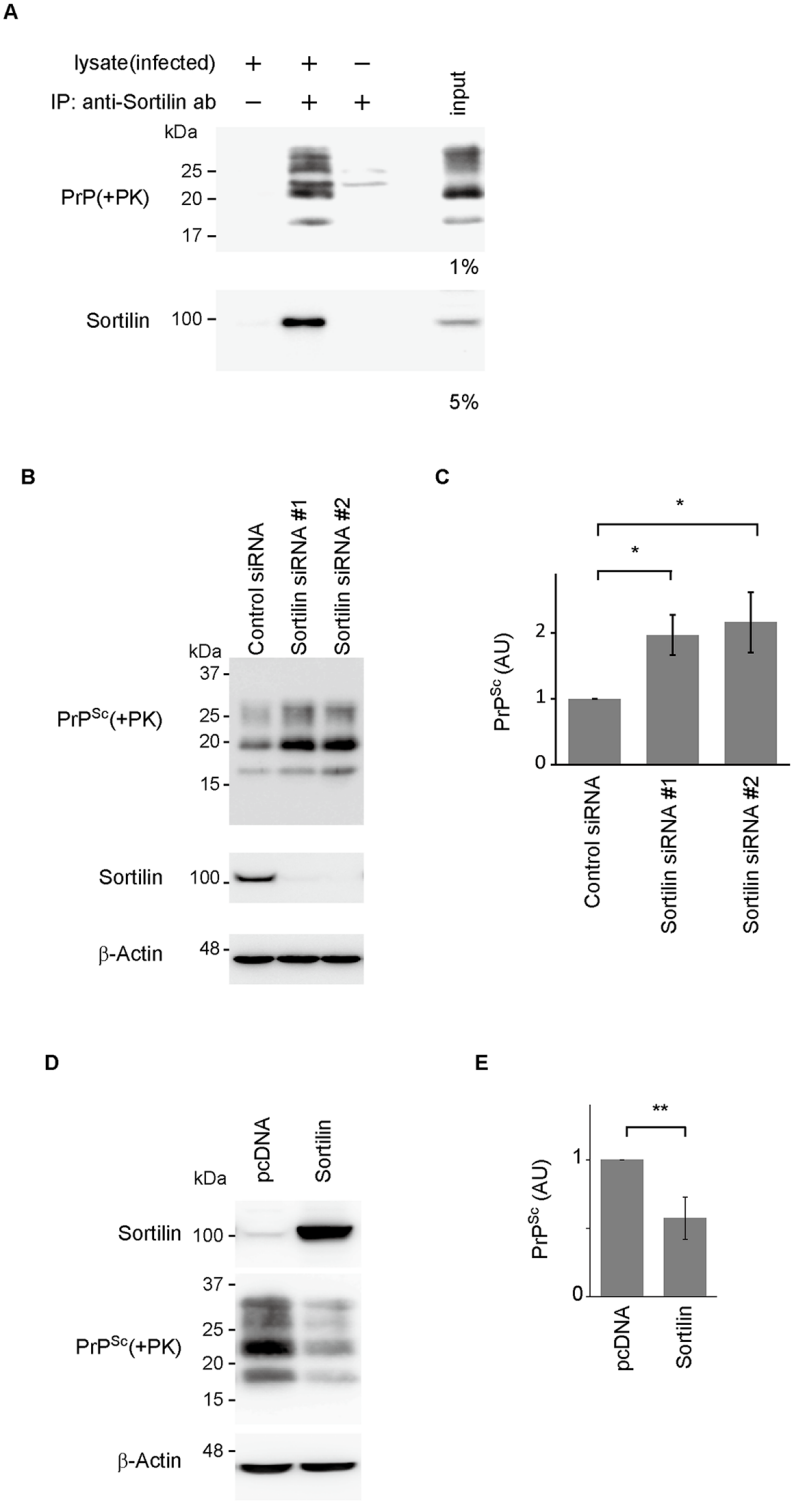
Sortilin regulates PrP^Sc^ levels in prion-infected cells. (A) Protein interaction assay using protein-G-conjugated magnetic beads in 22L-infected N2aC24L1-3 cells with rabbit polyclonal anti-sortilin Abs. The immunocomplexes were digested with proteinase K (PK) (100 μg/mL) at 37°C for 1 h and subjected to Western blotting for PrP^Sc^. Sortilin was also detected by Western blotting. (B) Western blotting for PrP^Sc^ and sortilin in N2aC24L1-3 cells 4 days after transfection with control and sortilin-specific siRNAs. (C) Quantification of PrP^Sc^ in (B) after normalization against β-actin. Each signal intensity in Sort-KD cells was evaluated against that in control cells. * p < 0.05. (D) Western blotting for PrP^Sc^ and sortilin in N2aC24L1-3 cells 3 days after transfection with control pcDNA or pcDNA-Sortilin. (E) Quantification of PrP^Sc^ in (D) after normalization against β-actin. Signal intensity in sortilin-overexpressing cells (Sortilin) was evaluated against that in control cells (pcDNA). Data are means ± SD of 3 independent experiments. ** p < 0.01.

We also assessed the role of sortilin in the degradation of PrP^Sc^. Protein interaction assay using protein-G-conjugated magnetic beads in prion-infected N2aC24L1-3 cells showed that PrP^Sc^ and sortilin were co-collected by anti-sortilin Abs (Fig 5A), suggesting that sortilin interacts with PrP^Sc^. siRNA-mediated knockdown of sortilin increased PrP^Sc^ in N2aC24L1-3 cells (Fig 5B and 5C). In contrast, overexpression of sortilin reduced PrP^Sc^ in N2aC24L1-3 cells (Fig 5D and 5E). These results indicate that sortilin could also be involved in PrP^Sc^ degradation.

**Fig 6:**
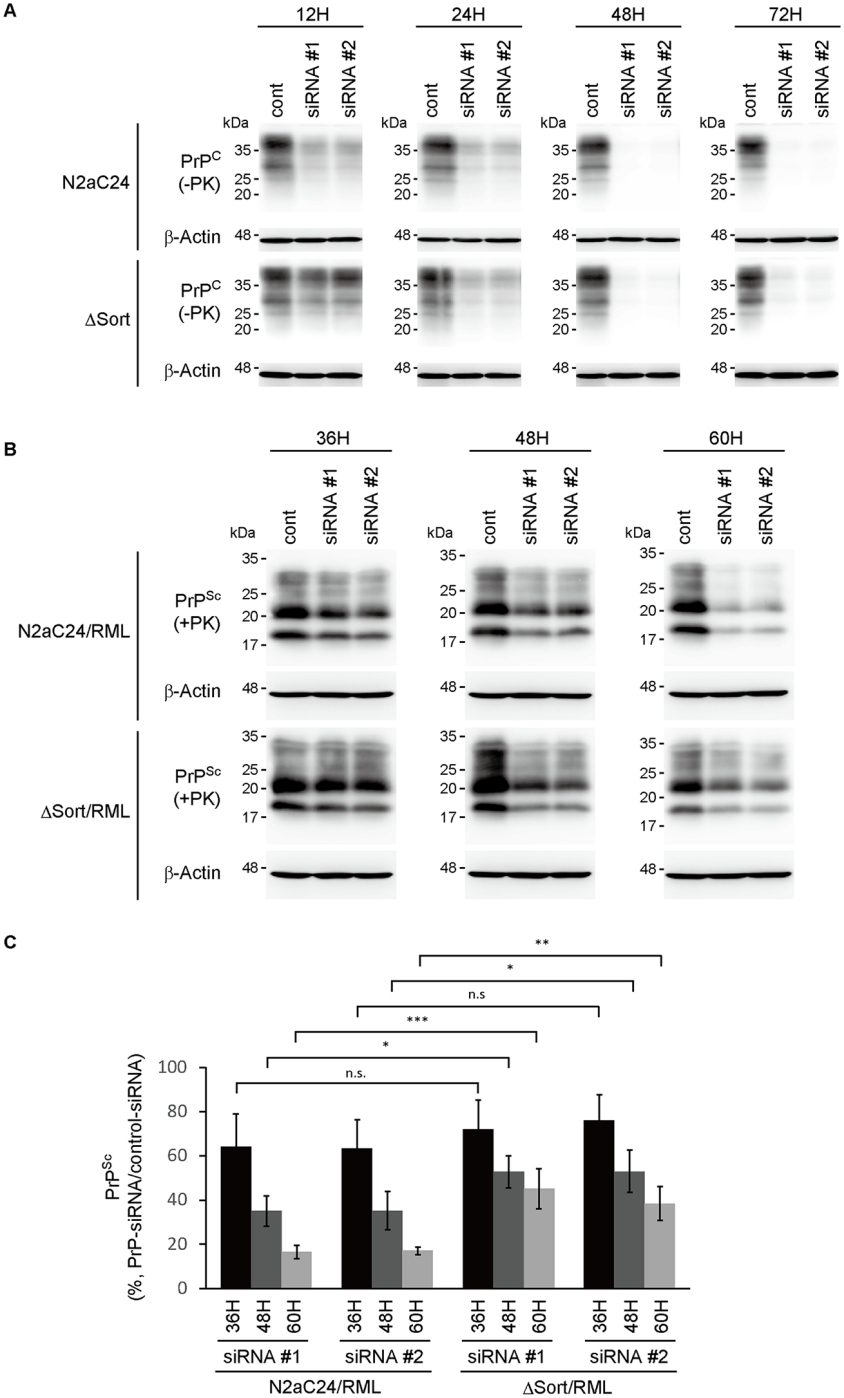
PrP^Sc^ degradation is delayed in sortilin-knockdown cells infected with RML prions. (A) Western blotting for PrP^C^ in N2aC24 and ΔSort#1 cells at 12, 24, 48 and 72 h after transfection with control and PrP-specific siRNAs. (B) Western blotting for PrP^Sc^ in RML-infected N2aC24 (N2aC24/RML) and RML-infected ΔSort#1 (ΔSort/RML) cells 36, 48 and 72 h after transfection with control and PrP-specific siRNAs. (C) Quantification of PrP^Sc^ in (B) after normalization against β-actin. Signal intensities in each lane in PrP-knockdown cells were evaluated against that in control siRNA-transfected cells in each blot. Data are means ± SD of 3 independent experiments. n.s., not significant; * p < 0.05, ** p < 0.01, *** p < 0.001.

We then evaluated the degradation kinetics of PrP^Sc^ in prion-infected cells with or without sortilin. For this study, it is important to prevent the *de novo* production of PrP^Sc^ from PrP^C^. PrP siRNA#1 and #2 reduced PrP^C^ in N2aC24 and ΔSort#1 cells from 24 h after transfection, to less than 10% of that in control siRNA-transfected N2aC24 and ΔSort#1 cells (Fig 6A). These results indicate that the *de novo* production of PrP^Sc^ from PrP^C^ could be negligible from 24 h after transfection with PrP siRNAs in these cells even after infection with prions. We thus investigated PrP^Sc^ in RML-infected N2aC24 (N2aC24/RML) and ΔSort#1 (ΔSort/RML) cells at 36, 48 and 60 h after transfection with PrP siRNAs. Control siRNA did not affect PrP^Sc^ levels in these cells (S11A and S11B Fig). However, PrP^Sc^ was decreased after transfection with PrP siRNAs (Fig 6B). In N2aC24/RML cells 60 h after transfection with PrP siRNAs, PrP^Sc^ was reduced to less than 20% of that in control siRNA-transfected N2aC24/RML cells (siRNA#1, 16.5%; siRNA#2, 17.1%) (Fig 6B and 6C). However, significantly higher levels of PrP^Sc^ were still observed in ΔSort/RML cells 60 h after transfection with PrP siRNAs (siRNA#1, 45.2%; siRNA#2, 38.5%) (Fig 6B and 6C). Similar results were obtained in 22L prion-infected N2aC24 and ΔSort#1 cells (S11C and S11D Fig). These results indicate that sortilin is also involved in the degradation of PrP^Sc^.

### Prion infection cell-autonomously reduces sortilin after PrP^Sc^ production

**Fig 7:**
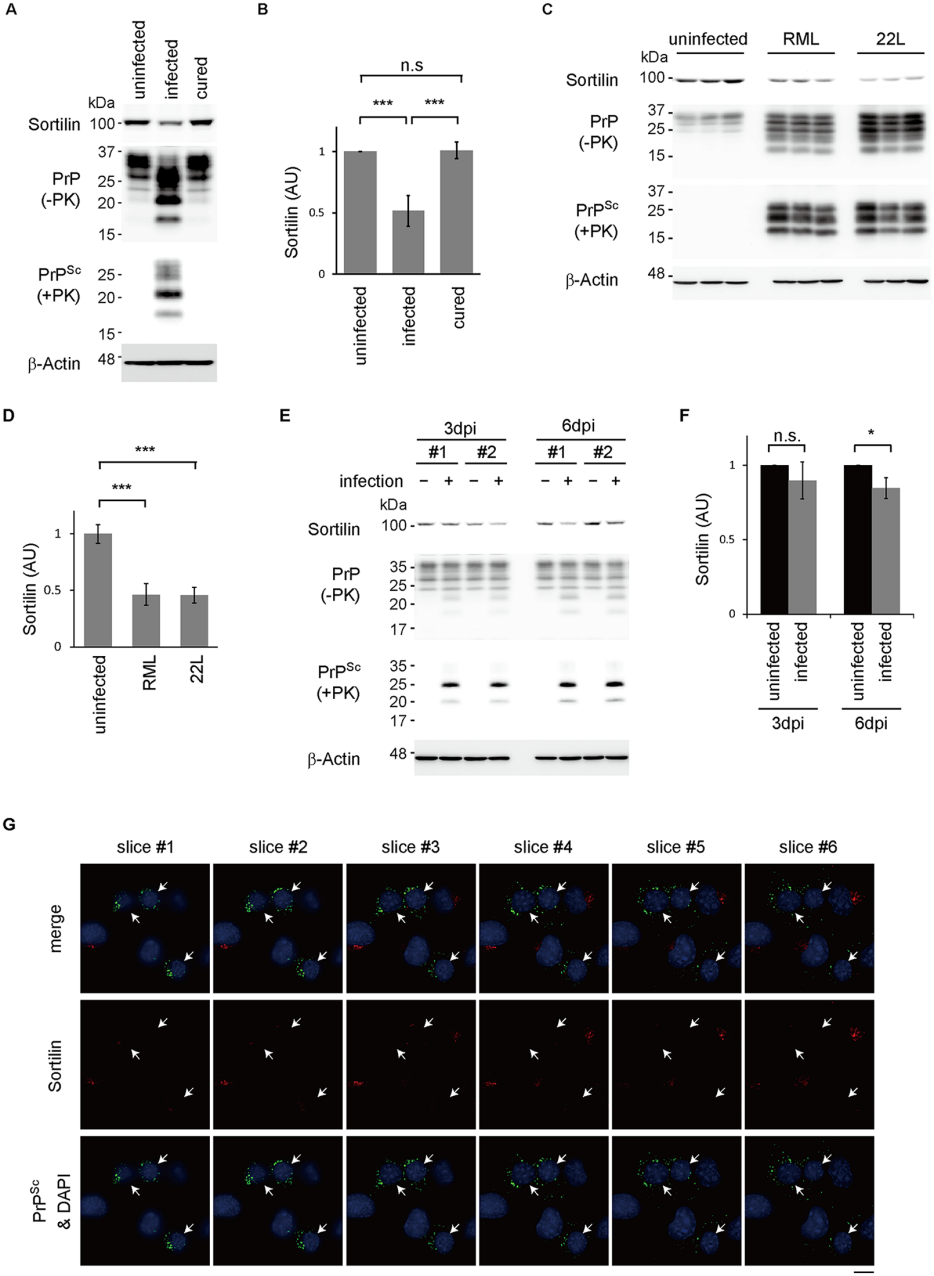
Prion infection decreases sortilin in a cell-autonomous way. (A) Western blotting for sortilin in N2aC24, N2aC24L1-3, and cured N2aC24L1-3 cells. PrP^C^ and PrP^Sc^ were also detected by Western blotting. (B) Quantification of sortilin in (A) after normalization against β-actin. The signal intensities in infected and cured cells were evaluated against those in uninfected cells. Data are means ± SD of 3 independent experiments. n.s., not significant; *** p < 0.001. (C) Western blotting for sortilin in the brains of mice infected with or without RML and 22L prions. PrP^C^ and PrP^Sc^ were also detected by Western blotting. (D) Quantification of sortilin in (C) after normalization against β-actin. The signal intensity in RML or 22L-infected brains was evaluated against that in uninfected brains. Data are means ± SD of 3 independent brains. *** p < 0.001. (E) Western blotting for sortilin in N2aC24 cells 3 and 6 days after freshly infected with RML prions. PrP^C^ and PrP^Sc^ were also detected by Western blotting. (F) Quantification of sortilin in (E) after normalization against β-actin. Signal intensity in infected cells was evaluated against that in uninfected cells. Data are means ± SD of 3 independent experiments. n.s., not significant; * p < 0.05. (G) Double immunofluorescent staining of sortilin (red) and PrP^Sc^ (green) in N2aC24 cells 9 days after being freshly infected with RML prions. DAPI was used for nuclear stain (blue). Six serial vertical images of the cells with 1 μm intervals are shown. Arrows indicate PrP^Sc^-positive cells. Bar, 10 μm.

We then asked if prion infection could affect sortilin. Interestingly, sortilin was reduced in N2aC24L1-3 cells to 52% of that in N2aC24 cells (Fig 7A and 7B). However, other VPS10P molecules were not reduced (S12A and S12B Fig). The reduced levels of sortilin were recovered in cured N2aC24L1-3 cells, which were cured from prion infection after treatment with SAF32 anti-PrP Ab, to that in N2aC24 cells (Fig 7A and 7B). Sortilin mRNA was similarly expressed between N2aC24 and N2aC24L1-3 cells (S12C Fig). ScN2a cells, N2a cells persistently infected with RML prions, also expressed sortilin less than N2a cells (S12D and S12E Fig). Moreover, sortilin was reduced in the brains of terminally ill mice infected with RML and 22L prions to 46.3 and 45.6%, respectively, compared to uninfected mouse brains (Fig 7C and 7D). These results indicate that prion infection could reduce sortilin.

We then treated uninfected N2aC24 and infected N2aC24L1-3 cells with inhibitors to lysosomes (NH_4_Cl and concanamycin A) or proteasomes (MG132). Treatment with NH_4_Cl or concanamycin A increased sortilin in uninfected cells (S13A–S13D Fig). However, sortilin was much more increased in infected cells after treatment with NH_4_Cl and concanamycin A (S13A–S13D Fig). In contrast, MG132 did not affect sortilin levels (S13A and S13B Fig). These results suggest that sortilin could be degraded in lysosomes, and that the lysosomal degradation of sortilin could be stimulated in prion-infected cells.

We also monitored the levels of sortilin in N2aC24 cells freshly infected with RML prions. There was no significant decrease in sortilin by 3 days post-infection (dpi) while PrP^Sc^ was obviously detectable (Fig 7E and 7F). Sortilin was decreased at 6 dpi (Fig 7E and 7F). These results suggest that prion infection reduces sortilin, and that the sortilin reduction is preceded by PrP^Sc^ production. We also performed double immunofluorescence staining for sortilin and PrP^Sc^ in freshly infected N2aC24 cells at 9 dpi. PrP^Sc^ was specifically stained using the mAb132, which was demonstrated to specifically visualize PrP^Sc^ under partially denatured conditions [21]. Since the subcellular positions of sortilin and PrP^Sc^ might differ vertically in infected cells, 6 horizontally serial images at 1 μm interval were used to detect sortilin and PrP^Sc^. In cells displaying green fluorescence for PrP^Sc^, little or no sortilin (red fluorescence) was detected in any slices (Fig 7G). In contrast, bright red fluorescence for sortilin was observed only in the cells negative for PrP^Sc^ (Fig 7G). These results indicate that prion infection could reduce sortilin in a cell-autonomous fashion after PrP^Sc^ accumulation.

Sortilin is known to interact with and transport Trk receptors to the cell surface, thereby enhancing nerve growth factor (NGF) signaling leading to activation of MAP kinases [11]. To investigate whether or not prion infection could affect the function of sortilin, we stimulated uninfected N2aC24 and prion-infected N2aC24L1-3 cells with NGF. Phosphorylated ERK1/2 was increased in N2aC24 and N2aC24L1-3 cells after stimulation (S14A–S14C Fig). However, the levels of phosphorylated ERK1/2 were significantly lower in N2aC24L1-3 cells than in N2aC24 cells (S14A–S14C Fig). These results indicate that NGF signaling is impaired in N2aC24L1-3 cells, suggesting that sortilin might be functionally disturbed in prion-infected cells.

### Prion disease is aggravated in sortilin-KO mice after infection with prions

**Fig 8:**
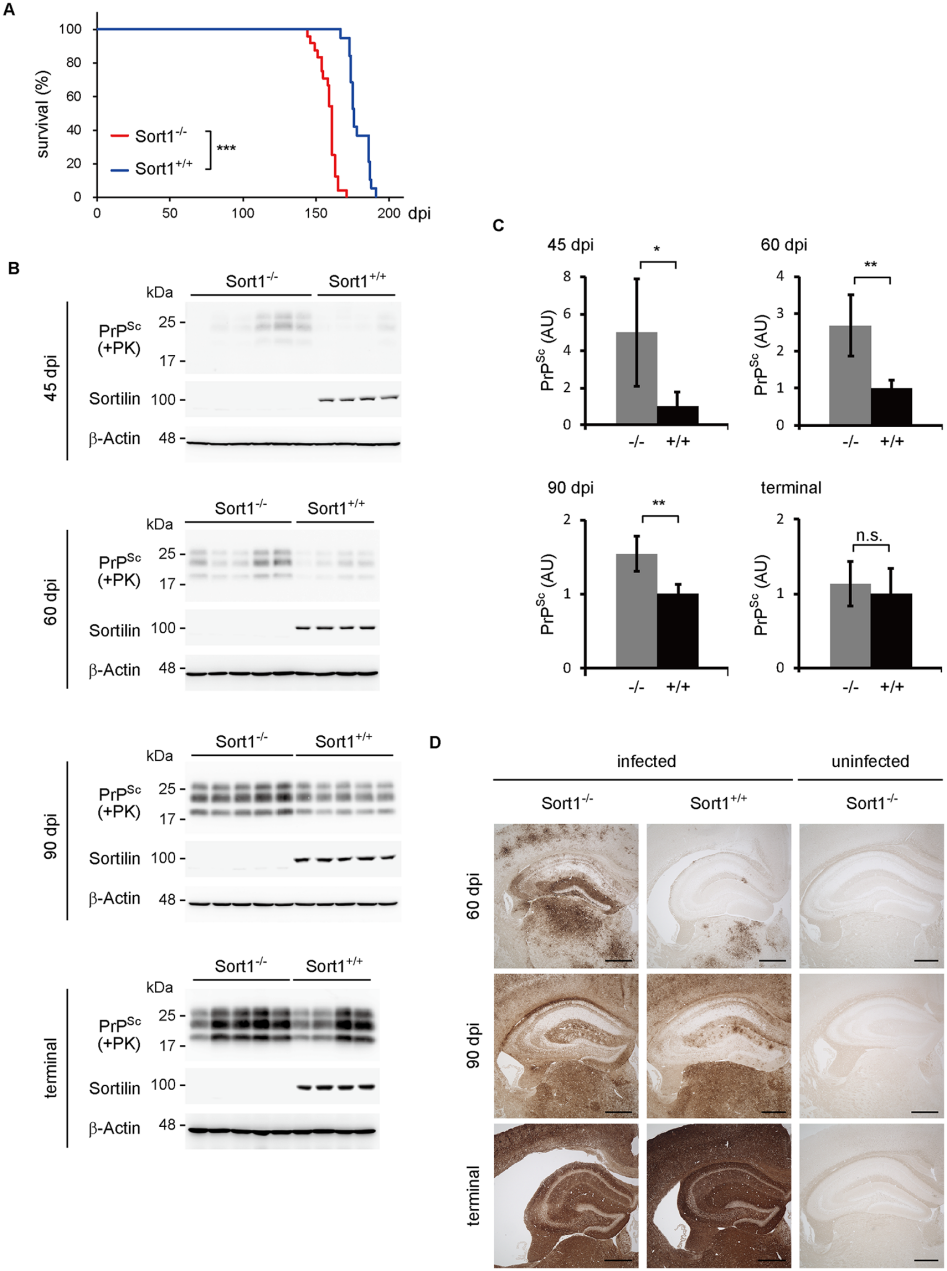
Sortilin-KO mice have accelerated prion disease with earlier accumulation of PrP^Sc^ in their brains. (A) Kaplan-Meier survival curves for Sort1^-/-^ and Sort1^+/+^ female mice inoculated with RML prions. Data was statistically analyzed using the log-lank test (Sort^-/-^, n = 24; Sort^+/+^, n = 19). *** p < 0.001. (B) Western blotting for PrP^Sc^ in the brains of Sort1^-/-^ and Sort1^+/+^ female mice 45, 60, 90 dpi and at terminal stage. Sortilin was also detected by Western blotting. (C) Quantification of PrP^Sc^ in (B) after normalization against β-actin. Signal intensity in Sort1^-/-^ mice was evaluated against that in Sort1^+/+^ mice. Data are means ± SD of 4–5 independent brains. n.s, not significant; * p < 0.05, ** p < 0.01. (D) Immunohistochemical staining of PrP^Sc^ in the hippocampus of Sort1^-/-^ and Sort1^+/+^ female mice at 60 and 90 dpi and at terminal stage. PBS-inoculated mice brain hippocampus was also stained as a control. The brains removed from PBS-inoculated Sort1^-/-^ mice aged 16, 20, and 30 weeks were used as controls for 60 and 90 dpi and terminal stage, respectively. Bar, 300 μm.

We then evaluated the effects of sortilin deficiency on the pathogenesis of prion disease using sortilin-KO (Sort1^-/-^) mice. Sort1^-/-^ mice were viable and fertile and showed no gross abnormalities [11,22]. Sort1^+/+^ (n = 19) and Sort1^-/-^ female mice (n = 24) were intracerebrally inoculated with RML prions. Incubation and survival times were significantly shortened in Sort1^-/-^ mice (Fig 8A, S1 Table). Sort1^-/-^ and Sort1^+/+^ mice developed symptoms at 150.9 ± 7.8 and 171.9 ± 6.0 dpi, respectively (Fig 8A, S1 Table). Western blotting also showed earlier accumulation of PrP^Sc^ in the brains of infected Sort1^-/-^ mice. PrP^Sc^ was scarcely detectable in the brains of Sort1^+/+^ mice at 45 dpi (Fig 8B and 8C). However, it was obvious in Sort^-/-^ mice at 45 dpi (Fig 8B and 8C). PrP^Sc^ levels were still significantly higher in Sort1^-/-^ mice than in Sort1^+/+^ mice at 60 and 90 dpi (Fig 8B and 8C). However, no difference in the levels of PrP^Sc^ was observed between Sort1^+/+^ and Sort1^-/-^ mice at terminal stage (Fig 8B and 8C). Immunohistochemical analysis of the brains of infected Sort1^+/+^ and Sort1^-/-^ mice for PrP^Sc^ showed consistent results. PrP^Sc^ was detectable in a much larger area of the brains of Sort1^-/-^ mice, compared to that in Sort1^+/+^ mice, at 60 dpi (Fig 8D). However, PrP^Sc^ became indistinguishably accumulated throughout the brains of Sort1^-/-^ and Sort1^+/+^ mice at terminal stage (Fig 8D). Similar results were also obtained with Sort1^-/-^ and Sort1^+/+^ male mice inoculated with RML prions (S15A–S15D Fig, S2 Table). These results show that sortilin deficiency accelerates prion disease by causing early accumulation of PrP^Sc^ in the brains of mice after infection with prions, reinforcing the inhibitory role of sortilin in the pathogenesis of prion disease.

## Discussion

In the present study, we showed that PrP^Sc^ accumulation could be enhanced through PrP^Sc^-stimulated degradation of sortilin, a member of the VPS10P sorting receptor family. Sortilin functions as a negative regulator for PrP^Sc^ accumulation by sorting PrP^C^ and PrP^Sc^ to the late endosome/lysosome protein degradation pathway. However, PrP^Sc^ stimulates sortilin to be degraded in lysosomes, thereby disturbing the inhibitory role of sortilin and eventually leading to the further accumulation of PrP^Sc^.

**Fig 9:**
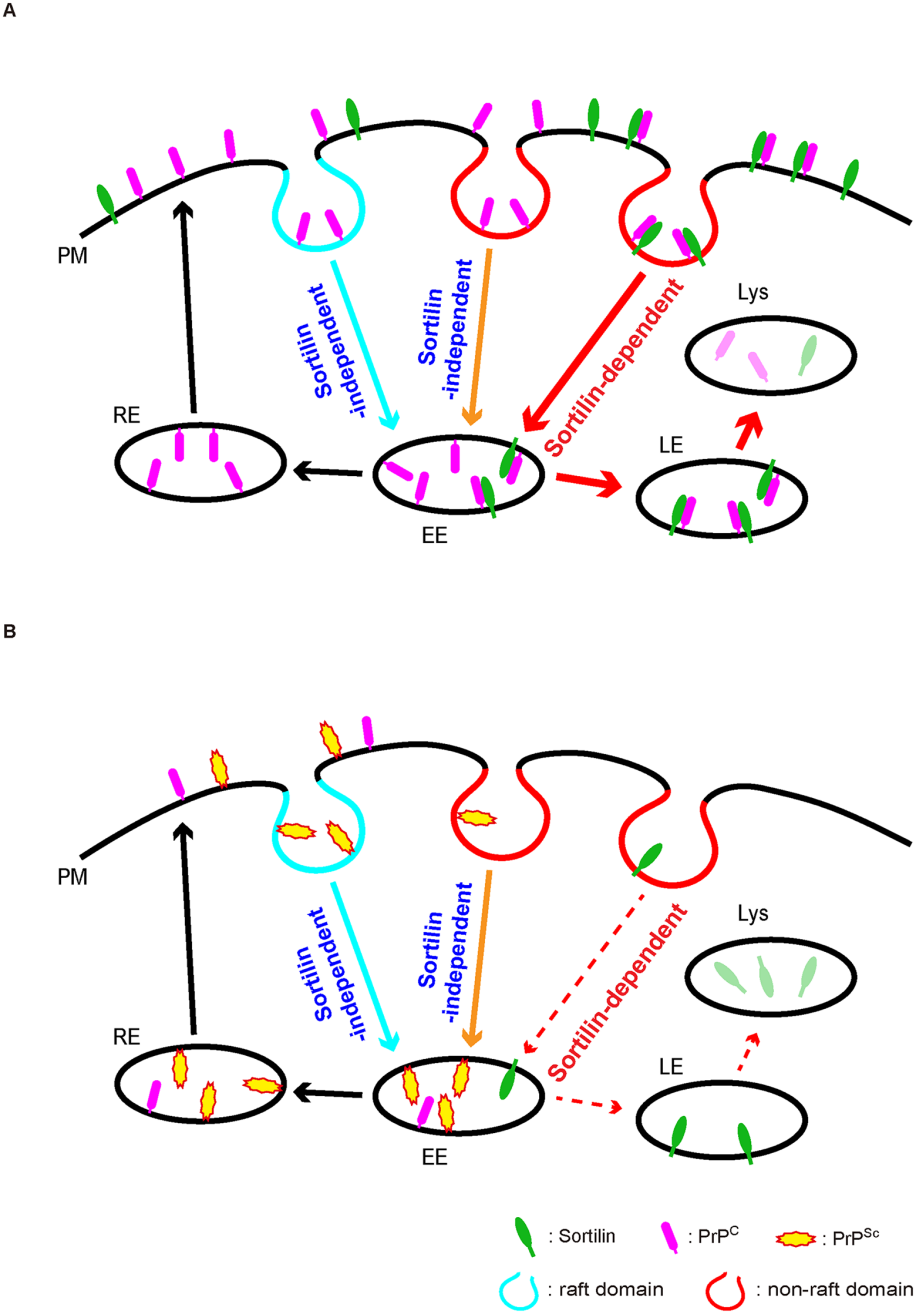
A possible amplification model of PrP^Sc^ in prion infected cells. (A) Sortilin-dependent and -independent endocytosis of PrP^C^ in uninfected cells. A portion of PrP^C^ is endocytosed with sortilin and trafficked to lysosomes for degradation. Others are trafficked either to lysosomes for degradation or to the recycling pathway in a sortilin-independent way. (B) Endocytosis of PrP^C^ and PrP^Sc^ in prion-infected cells. Prion infection stimulates lysosomal degradation of sortilin, thereby impairing the sortilin-mediated trafficking of PrP^C^ and PrP^Sc^ to lysosomes. As a result, PrP^C^ and PrP^Sc^ are increased at the raft domains and endocytosed via the sortilin-independent pathway, increasing the conversion of PrP^C^ into PrP^Sc^. The decreased degradation of PrP^Sc^ and the increased conversion of PrP^C^ into PrP^Sc^ both contribute to the constitutive production of PrP^Sc^ in prion-infected cells. Dashed arrows indicate restricted trafficking. EE: early endosomes, RE: recycling endosomes, LE: late endosomes, Lys: lysosomes, PM: plasma membrane.

PrP^C^ is a GPI-anchored membrane protein located in raft domains and, to a lesser extent, in non-raft domains. Some of the PrP^C^ molecules internalized are delivered back to the cell surface directly or indirectly via the recycling endosome compartments and others are transported to lysosomes for degradation [7] (Fig 9A). We showed that sortilin could directly interact with PrP^C^ on the cell surface through the VPS10P domain through the residues 610–753 encompassing cysteine rich 10CCs [23] of sortilin and the N-terminal residues 23–88 of PrP^C^. Sortilin-knockdown increased PrP^C^ on the cell surface and reduced the localization of PrP^C^ to lysosomes, suggesting that the increased surface expression of PrP^C^ in sortilin-knockdown cells might be caused by the decreased trafficking of PrP^C^ to lysosomes for degradation. Sortilin was predominantly located in non-raft domains, and PrP^C^ accumulated at raft domains and decreased in non-raft domains in sortilin-KO cells. It is thus conceivable that sortilin could function to retain PrP^C^ in non-raft domains and be involved in trafficking of non-raft surface PrP^C^ to lysosomes for degradation (Fig 9A). Low-density lipoprotein receptor-related protein 1 has also been reported as a candidate cargo receptor for the non-raft PrP^C^ [24]. A sortilin-independent pathway may also play a role in PrP^C^ internalization (Fig 9A). We showed that sortilin deficiency increased PrP^C^ at raft domains and shifted PrP^C^ internalization into the recycling endosomes from lysosomes in cells. It is thus conceivable that the internalization of raft PrP^C^ to the recycling endosomes could be sortilin-independent (Fig 9A). Taken together, these results suggest that PrP^C^ located in non-raft domains could be internalized to lysosomes for degradation partly via the sortilin-dependent pathway while an internalization pathway to direct PrP^C^ from raft domains to the endocytic recycling pathway could be sortilin-independent (Fig 9A).

Sortilin-knockdown increased PrP^Sc^ in prion-infected cells. In contrast, overexpression of sortilin decreased PrP^Sc^. Moreover, sortilin-KO mice developed the disease earlier than wild-type mice after intracerebral inoculation with RML prions, with earlier accumulation of PrP^Sc^ in their brains. These results indicate that sortilin is a negative regulator for PrP^Sc^ accumulation. Raft domains may be a site for the conversion of PrP^C^ into PrP^Sc^ [25], although the exact site of PrP^Sc^ production remains controversial. Sortilin could retain surface PrP^C^ at non-raft domains and transport it to lysosomes for degradation, thereby reducing PrP^C^ located in raft domains. It is thus possible that the reduction of PrP^C^ in raft domains by sortilin could delay the conversion of PrP^C^ into PrP^Sc^, eventually leading to less accumulation of PrP^Sc^. Sortilin also interacts with PrP^Sc^. Kinetics studies for PrP^Sc^ showed that knockout of sortilin delayed PrP^Sc^ clearance in prion-infected cells. Thus, sortilin also could function to sort PrP^Sc^ for degradation, reducing PrP^Sc^ accumulation. Some PrP^Sc^ molecules are trafficked to lysosomes for degradation via the endolysosomal pathway from the cell surface [21,26,27]. Others are retrogradely transported to the Golgi apparatus where they are subjected to Golgi quality control and trafficked to lysosomes for degradation [28]. Sortilin is expressed on the cell surface and the Golgi apparatus [29]. Therefore, sortilin might be involved in both degradation trafficking pathways of PrP^Sc^. However, a large portion of sortilin and PrP^Sc^ molecules differed in their membrane microdomain localization on the cell surface. Sortilin was predominantly detected in non-raft domains. In contrast, PrP^Sc^ was exclusively located in raft domains. It is thus likely that the sortilin-mediated lysosomal degradation of PrP^Sc^ from the cell surface through their direct interaction might be, if any, a minor event. We previously reported that PrP^Sc^ was abundantly detected in the recycling endosomes of prion-infected cells, suggesting that PrP^Sc^ molecules accumulated in the recycling endosomes also might not be directly affected by sortilin.

Sortilin was reduced in both prion-infected cultured cells and mouse brains. Sortilin mRNA was not decreased in prion-infected cells. Reduction of sortilin in prion-infected cells was recovered by treatment with lysosomal inhibitors but not proteasomal inhibitor, suggesting that prion infection could stimulate degradation of sortilin in lysosomes. Immunofluorescent staining of freshly infected cells revealed that sortilin was barely detectable in PrP^Sc^-positive cells but abundant in PrP^Sc^-negative cells. PrP^Sc^ accumulation preceded the reduction of sortilin. These results suggest that PrP^Sc^ accumulated after prion infection could cause sortilin degradation in lysosomes in a cell-autonomous fashion, and that the enhanced degradation of sortilin could abrogate the negative role of sortilin in PrP^Sc^ accumulation in prion-infected cells. Thus, sortilin-mediated sorting of PrP^C^ and PrP^Sc^ to lysosomes for degradation could be disturbed in prion-infected cells, causing an increase in PrP^C^ and PrP^Sc^ and eventually leading to progressive accumulation of PrP^Sc^ (Fig 9B). In prion-infected cells, due to the disturbed function of sortilin, PrP^C^ might also be increasingly located in raft domains, where PrP^C^ is postulated to efficiently convert into PrP^Sc^ [25], and might be internalized into the recycling endosomes, where PrP^Sc^ was reported to be abundantly detectable [26] (Fig 9B). Increased localization of PrP^C^ in raft domains and in the recycling endosomes in prion-infected cells also could contribute to progressive accumulation of PrP^Sc^ by increasing the conversion of PrP^C^ into PrP^Sc^. Elucidation of the mechanism by which PrP^Sc^ accumulation stimulates the lysosomal degradation of sortilin would be helpful to further understanding of the mechanism for the progressive accumulation of PrP^Sc^.

Sortilin also regulates neuronal cell viability by controlling the release of pro- and matured-form of neurotrophins (NTs), such as NGF and brain-derived neurotrophic factor, as does the transport of their receptors, TrkA, TrkB, and TrkC, to the plasma membrane [9]. No gross abnormalities were reported in Sort1^-/-^ [11,22], probably due to compensatory mechanisms of other family molecules. However, cultured dorsal root ganglion neurons lacking sortilin showed impaired neurite outgrowth upon NGF stimulation [11]. Loss of sortilin also aggravated neurological phenotypes observed in p75 NT receptor (p75^NTR^)-KO mice [11]. Sortilin also acts as a co-receptor of p75^NTR^ against proNTs to transduce cell death signals [30]. We found that sortilin was significantly reduced in prion-infected cells and mouse brains, and that the NGF signaling was disturbed in prion-infected cells. It might thus be interesting to investigate whether or not the sortilin-mediated NT signals are involved in the pathogenesis of prion disease.

It remains controversial whether or not degradation of sortilin might be stimulated in lysosomes in other neurodegenerative diseases. Reduced levels of sortilin have been reported in the limbic and occipital regions of AD brains [31]. To the contrary, increased expression of sortilin has been demonstrated in the temporal cortex of AD brains [32]. Other investigators showed no alteration of sortilin levels in the superior frontal and superior temporal cortices of AD brains [33].

In short, we presented a novel accumulation mechanism of PrP^Sc^ through degradation of sortilin. Prion infection stimulated degradation of sortilin in lysosomes, reducing sortilin levels in prion-infected cells. The reduction of sortilin disturbs its function to sort PrP^C^ and PrP^Sc^ to the late endosomal/lysosomal compartments for degradation. As a result, PrP^C^ is increasingly converted to PrP^Sc^ and PrP^Sc^ degradation is delayed, and eventually PrP^Sc^ progressively accumulates in prion-infected cells. Thus, accelerating the sortilin-mediated lysosomal degradation of PrP^C^ and PrP^Sc^ might be therapeutic in prion diseases.

## Materials and methods

### Ethics statement

All animal experiments complied with Japanese legislation (Act on Welfare and Management of Animals, 1973, revised in 2012). The Ethics Committee of Animal Care and Experimentation of Tokushima University approved the animal experiments in this study (approval number T27-102). Animals were cared for in accordance with The Guiding Principle for Animal Care and Experimentation of Tokushima University and guidelines under the jurisdiction of the Ministry of Education, Culture, Sports, Science and Technology, Japan (Fundamental Guidelines for Proper Conduct of Animal Experiment and Related Activities in Academic Research Institutions, 2006).

### Abs

The antibodies used in this study are as follows: rabbit anti-sortilin Abs (12369-1AP, Proteintech, Rosemont, IL), goat anti-sortilin Abs (AF2934, R&D systems, Minneapolis, MN), mouse monoclonal anti-sortilin Ab (clone 48, BD Bioscience, San Jose, CA), sheep anti-SorLA Abs (AF5699, R&D systems), goat anti-SorCS1 Abs (AF3457, R&D systems), sheep anti-SorCS2 Abs (AF4237, R&D systems), rat anti-SorCS3 Abs (MAB3067, R&D systems), rat anti-LAMP1 Ab (ab25245, Abcam, UK), rabbit anti-Rab9 Abs (EPR13272, Abcam), rabbit anti-Rab11 Abs (5589, Cell signaling, MA, USA), rabbit anti-flotillin-2 Abs (3436, Cell signaling), mouse anti-β-actin Ab (A5316, Sigma-Aldrich, St. Louis, MO), 6D11 mouse anti-PrP Ab (SIG-39810, Biolegend, San Diego, CA), SAF61 mouse anti-PrP Ab (A03205, Bertin pharma, Montigny le Bretonneux, France), SAF83 mouse anti-PrP Ab (A03207, Bertin pharma), rabbit anti-PrP Abs (18635, Immuno Biological Laboratories, Gunma, Japan), rabbit anti-TSG101 Ab (14497-1-AP, ProteinTech, IL, USA), rabbit anti-flottilin-1 Ab (Ab 41927, Abcam), mouse anti-GM130 Ab (610823, Transduction Laboratories, CA, USA), rabbit anti-Bcl-2 Ab (#3498, Cell Signaling), mouse anti-EEA1 Ab (610456, Transduction Laboratories), mouse anti-Erk1 Ab (610456, BD Biosciences), mouse anti-pErk1/2 Ab (612358, BD Biosciences), Alexa Fluoro 488-conjugated anti-mouse IgG Abs (Thermo Fisher Scientific, Rockford, IL), Alexa Fluoro 546-conjugated anti-goat IgG Abs (Thermo Fisher Scientific), Alexa Fluoro 546-conjugated anti-rat IgG Abs (Thermo Fisher Scientific), Alexa Fluoro 488-conjugated anti-goat IgG Abs (Thermo Fisher Scientific). Mouse anti-PrP Ab clone 132 was kindly gifted from Prof Horiuchi, Hokkaido University [21].

### Plasmid construction and recombinant proteins

A mouse sortilin cDNA fragment was amplified from mouse brain QUICK-clone cDNA (Clontech, California, USA) by using PCR (primers; 5’-cctctcgagatggagcggccccggggagct-3’, 5’-ctcaagcttctattccaggaggtcctcatctga-3’) and the amplified fragment was subcloned into pcDNA3.1(-) (Invitrogen) to make an expression vector for full-length sortilin, designated as pcDNA-Sortilin. DNA fragments encoding the VPS10P domain of sortilin, which corresponds to residues 76–750, (primers; 5’-cctctcgagggcgcgcccgccgaggaccaa-3’, 5’-ctcaagcttctaggaattctgctttgtggg-3’) were also amplified by PCR using pcDNA-Sortilin as a template and subcloned into pGEX4T-2 (GE healthcare, Little Chalfont, UK) to express glutathione S-transferase (GST)-tagged VPS10P domain of sortilin in *E coli*. GST-tagged VPS10P domain and GST were induced in *E*. *coli* with 0.1 mM isopropyl thiogalactoside at 37°C for 3 h and purified with glutathione-beads. His-tagged recombinant full-length mouse PrP and PrP without residues 23–88 were prepared as previously described [34].

For construction of an expression vector, termed pEF1-moPrP(3F4), encoding full-length mouse PrP with a 3F4 tag, the *Bam*H I/*Xba* I-digested fragment of pcDNA3.1-moPrP(3F4) [35] was inserted into *Bam*H I/*Xba* I-digested pEF1/Myc-His (Invitrogen). To construct an expression vector, pEF1-moPrP(3F4)Δ23–88, encoding 3F4-tagged mouse PrP with residues 23–88 deleted and the 5’ fragment of moPrP(3F4)Δ23–88 cDNA was amplified by PCR using pcDNA3.1-moPrP(3F4) as a template with a BamHI-PrP(ATG)-S sense primer (5’-tcggatcccgtcatc**atg**gcgaac-3’; underlined, *Bam*H I site; bold, start codon) and a moPrP(3F4)Δ23–88 anti-sense primer (5’-cctccttggcc*gcagaggccga*-3’; underlined, residues 89–90; italic, residues 19–22). Then, full-length moPrP(3F4)Δ23–88 cDNA was amplified by PCR using pcDNA3.1-moPrP(3F4) as a template with the amplified 5’ fragment as a sense primer and a PrP(stop)-XbaI-AS anti-sense primer (5’-cctctagagc**tca**tcccacgatcag-3’; underlined, *Xba* I site; bold, stop codon). After sequence confirmation, the amplified fragment was inserted into *Bam*H I/*Xba* I-digested pEF1/Myc-His (Invitrogen).

For the construction of a Sortilin-mycHis expression vector, cDNA encoding full-length sortilin was amplified using pcDNA-Sortilin as a template by PCR with a primer pair of Sort-1 (5’-cctctcgag**atg**gagcggccccgggagct-3’; underlined, *Xho* I site; bold, start codon) and Sort-12 (5’-ctcaagcttttccaggaggtcctcatctga-3’; underlined, *Hind* III site) and inserted into *Xho* I/*Hind* III digested pcDNA3.1/mycHis(-)A (Invitrogen). Deletion mutants of sortilin were constructed as follows. For construction of sortilinΔ76–177, two DNA fragments with an overlapping DNA segment were amplified using pcDNA-Sortilin as a template by PCR with a primer pair of Sort-1 and Sort-D4 (5’-tatcacctttcc*gcggcgccaacggccagc*-3’; italic, residues 70–75; underlined, residues 178–183) and a primer pair of Sort-D3 (5’-cgttggcgccgc*ggaaaggtgatactaaca*-3’; underlined, residues 72–75; italic, residues 178–183) and Sort-12. These two fragments were hybridized at the overlapping DNA segment and subjected to PCR with a primer pair of Sort-1 and Sort-12, resulting in amplification of a DNA fragment encoding sortilinΔ76–177. The amplified fragment was inserted into *Xho* I/*Hind* III digested pcDNA3.1/mycHis(-)A (Invitrogen). Other DNA fragments encoding other sortilin mutants were similarly amplified and inserted into *Xho* I/*Hind* III digested pcDNA3.1/mycHis(-)A (Invitrogen). Sort-1 and Sort-D6 (5’-gaaggtttttcc*agagttctcaggaccaat*-3’; italic, residues 172–177; underlined, residues 291–294) and Sort-D5 (5’-cctgagaactct*ggaaaaaccttcaaaacc*-3’; underlined, residues 174–177; italic, residues 291–296) and Sort-12 were used for construction of sortilinΔ178–290. Sort-1 and Sort-D8 (5’-tatatagacccc*caagtcggatgttctcca*-3’; italic, residues 285–290; underlined, residues 410–413) and Sort-12 and Sort-D7 (5’-acatccgacttg*ggggtctatataacaagc*-3’; underlined, residues 287–290; italic, residues 410–415) were used for construction of sortilinΔ291–409. Sort-1 and Sort-D10 (5’-ggagtaaccccc*acggagggaagtcacgtt*-3’; italic, residues 404–409; underlined, residues 509–612) and Sort-D9 (5’-acttccctccgt*gggggttactcctgggcg*-3’; underlined, residues 406–409; italic, residues 509–514) and Sort-12 were used for construction of sortilinΔ410–508. Sort-1 and Sort-D12 (5’-atcctcttcaca*atcatctgagatgtacac*-3’; italic, residues 503–508; underlined, residues 610–613) and Sort-D11 (5’-atctcagatgat*tgtgaagaggatgactat*-3’; underlined, residues 505–508; italic, residues 610–615) and Sort-12 were used for construction of sortilinΔ509–609. Sort-1 and Sort-D14 (5’-aatagggacaga*attccgctcaaggatgtc*-3’; italic, residues 604–609; underlined, residues 754–757) and Sort-D13 (5’-cttgagcggaat*tctgtccctattatcctg*-3’; underlined, residues 606–609; italic, residues 754–759) and Sort-12 were used for construction of sortilinΔ610–753.

### Cell lines and animals

Cells were maintained at 37°C with 5% CO_2_ in air in Dulbecco’s Modified Eagle Medium (DMEM, Invitrogen, Carlsbad, CA) containing 10% fetal bovine serum (FBS). N2aC24, N2aC24L1-3, and cured N2aC24L1-3 cells were previously established elsewhere [26]. N2aC24 cells were cloned from mouse neuroblastoma N2a cells overexpressing exogenous mouse PrP^C^. N2aC24L1-3 cells were cloned from N2aC24 cells persistently infected with 22L scrapie prions. Cured N2aC24L1-3 cells are cured from prion infection by treatment with SAF32 anti-PrP Ab and then maintained in antibody-free DMEM with 10% FBS. ScN2a cells (kindly gifted from Prof Doh-ura, Tohoku University) were N2a cells persistently infected with RML scrapie prions.

Sortilin-KO cells, ΔSort#1 and #2 cells, were established using the CRISPR-Cas genome editing system. N2aC24 cells were transduced with pRGEN-Cas9-CMV (Takara bio, Shiga, Japan) and pRGEN_Mouse-Sort1_U6_SG_1 targeting the sequence (5’-cctgccgccgtcggccaggaccg-3’) (Takara bio), and subjected to limiting dilution cloning. Knockout of sortilin in ΔSort#1 and #2 cells was confirmed by Western blotting.

PrP-KO N2a cells, termed N2aΔPrP cells, were also established using the CRISPR-Cas genome editing system. N2a cells were transduced with pRGEN-Cas9-CMV (Takara bio) and pRGEN_PrP_U6_SG_1 targeting the sequence (5’-accggtggaagccggtatcccgg-3’) (Takara bio), and subjected to limiting dilution cloning. Knockout of PrP^C^ in N2aΔPrP cells was confirmed by Western blotting. To clone N2aΔPrP cells expressing full-length wild-type PrP^C^ or PrPΔ23–88, pEF1-moPrP(3F4) and pEF1-moPrP(3F4)Δ23–88 were linearized by *Sca* I and transfected into N2aΔPrP cells. The cells were treated with G418 and the G418-resistant cells were cloned by limiting dilution, resulting in establishment WT#1 and #2 cells and Δ23–88#1 and #2 cells.

Sortilin-KO (Sort1^-/-^) mice used in this study were previously produced elsewhere [11,22]. Sort1^-/-^ mice having been backcrossed for 10 generations into C57BL/6 were intercrossed with C57BL/6 mice (Charles River Laboratories, Kanagawa, Japan), and the resulting heterozygous mice (Sort^-/+^) were then intercrossed to obtain Sort^-/-^ and wild-type (Sort^+/+^) mice.

### Immunoprecipitation

Cells were lysed in buffer A [20 mM MES-KOH (pH 7.0), 0.15 M KCl, 1 mM DTT, 10% glycerol, 0.2% (w/v) CHAPS] containing protease inhibitor cocktail (Nakalai tesque, Kyoto, Japan). The lysate was cleared by centrifugation for 5 min at 20,000×g at 4°C and the supernatant was transferred to a new tube. 500 μL of supernatant containing 500 μg of total proteins were incubated with 1 μg of indicated Abs for 2 h. 5 μL of protein-G sepharose (GE healthcare) was added and the mixture was rotated for 4 h at 4°C. Thereafter, protein-G sepharose was precipitated and the precipitant was washed with buffer A 5 times. The final precipitate with protein-G sepharose was suspended in 50 μL of Laemmli’s sample buffer, heated, and subjected to Western blotting to detect proteins of interest with appropriate Abs.

### Protein interaction assay using Dynabeads protein-G

For detection of interaction of PrP^Sc^ and sortilin, 15 μL of Dynabeads protein-G (Thermo Fisher Scientific) was added instead of protein-G sepharose. Immunocomplexes were collected using magnet instead of centrifugation and washed with buffer A. The same procedure was repeated 5 times. The finally collected complexes were suspended in 45 μL of buffer B [150 mM NaCl, 50 mM Tris-HCl (pH 7.5), 0.5% (w/v) Triton X-100, 0.5% (w/v) sodium deoxycholate, 1 mM EDTA] containing 100 μg/ml proteinase K (PK) and incubated at 37°C for 60 min with mixing at 1,100 rpm using Thermomixer (Eppendorf, Hamburg, Germany). The PK-treated samples were denatured in Laemmli’s sample buffer and subjected to Western blotting to detect PrP^Sc^.

For detection of interaction of PrP^C^ and sortilin on the cell surface, cells were washed with ice-cold PBS. After blocking of the cells with buffer C [20 mM MES-KOH (pH 7.0), 0.15 M KCl, 0.25 M sucrose] containing 0.5% (w/v) bovine serum albumin (BSA) for 10 min at 4°C, the cell surface PrP was labeled with 1 μg/mL of SAF61 anti-PrP Ab in buffer C containing 0.5% (w/v) BSA for 30 min at 4°C. After washing the cells with ice-cold-PBS, they were suspended in 1 mL of buffer C. To homogenize the cells, the cell suspension was passed through a 27G needle 10 times. The homogenate was centrifuged at 500×g for 10 min at 4°C and the supernatant was incubated with 15 μL of Dynabeads protein-G with gentle agitation for 2 h at 4°C. The beads were collected by magnet and washed with buffer C. The collected immunocomplexes on the beads were washed with buffer A 5 times and finally suspended in 50 μL of Laemmli’s sample buffer and subjected to Western blotting.

### GST pulldown assay

2 μg of GST-tagged VPS10P domain of sortilin, which was pre-bound to 5 μL of glutathione beads, was incubated with 2 μg of His-tagged full-length recombinant PrP or His-tagged recombinant PrP lacking residues 23–88 in buffer A containing protease inhibitor cocktail for 2 h with rotation at 4°C. The precipitate was washed with buffer A 5 times, suspended in Laemmli’s sample buffer, heated, and subjected to Western blotting. GST-tagged VPS10P domain and His-tagged PrPs were detected with rabbit polyclonal anti-sortilin Abs and RGS-His Ab, respectively.

### Biotinylation of cell surface proteins

Biotinylation of cell surface proteins was carried out as described elsewhere [26]. In brief, cells (85–95% confluent) were washed with PBS and incubated with Sulfo-NHS-LC Biotin (Thermo Fisher Scientific) in PBS for 30 min at room temperature. The cells were then washed with 0.1 M glycine in PBS and lysed in buffer B. The lysate was mixed with NeutrAvidin UltraLink Resin (Thermo Fisher Scientific) for 4 h at 4°C and the biotinylated protein-resin complexes were collected by brief microcentrifugation. The complexes were then washed with the buffer and heated at 99°C for 10 min in Laemmli’s sample buffer to separate the biotinylated proteins from the complexes. The biotinylated proteins in the supernatant were subjected to Western blotting.

### Immunofluorescence staining

Cells were stained with indicated Abs as described previously [26]. In brief, cells grown on coverslips were fixed in 3% paraformaldehyde (PF) for 15min and treated with 0.1 M glycine in PBS for 10 min. Permeabilization was carried out using 0.1% Triton X-100 in PBS for 4 min at RT. To detect PrP^Sc^, the cells were treated with 5 M guanidinium thiocyanate for 10 min at RT. After washing with PBS, the cells were incubated with the first Ab in 5% FBS in PBS and then with fluorescent secondary Ab. For detection of PrP^Sc^, mouse anti-PrP Ab clone 132 [21] (kindly gifted from Prof Horiuchi, Hokkaido University) was used as a first Ab. After washing, the coverslips were mounted with Prolong Gold antifade reagent (Invitrogen). Fluorescence images were obtained using BIOREVO BZ-9000 (Keyence, Osaka, Japan), which is equipped with haze reduction function, which enables production of fluorescent images very similar to those taken by a confocal microscope. To assess the co-localization of proteins of interest, Pearson’s correlation coefficient was calculated using Co-localization Plugin (JaCoP) tool in Image J [36].

### Internalization assay of antibody-labeled surface PrP

Cells were washed with ice-cold PBS and treated with 1% BSA in PBS for 10 min at 4°C prior to incubation with the indicated anti-PrP Abs (1 μg/mL) for 10 min at 4°C in 1% BSA-containing PBS. The cells were then washed with ice-cold PBS and incubated at 37°C for 2 h. Thereafter, the cells were fixed with 3% paraformaldehyde, permeabilized with 0.1% Triton X-100, and stained with Alexa Fluoro 488 anti-mouse IgG Ab (Thermo Fisher Scientific). Fluorescent signals were observed using BIOREVO BZ-9000 (Keyence) and their intensities were analyzed using BZ-II analyzer (Keyence).

### Internalization assay of biotinylated surface PrP

Cells were washed three times with ice-cold PBS (pH7.4) and incubated with 5 mg/mL sulfo-NHS-SS-biotin (Thermo Fisher Scientific) in PBS (pH 7.4) at 4°C for 10 min to biotinylate cell surface proteins. After washing the cells twice with ice-cold PBS (pH 7.4) and incubation with 50 mM glycine in PBS (pH 7.4), the cells were further washed twice with ice-cold PBS (pH 7.4). Thereafter, the cells were incubated in DMEM medium at 37°C. After 2 h-incubation, the cells were washed three times with ice-cold PBS (pH 7.4), and biotin was removed from the proteins still on the cell surface by incubating the cells with 100 mM reducing glutathione in PBS (pH 7.4) at 37°C for 10 min. After washing the cells with ice-cold PBS (pH 7.4), the cells were lysed in buffer B containing protease inhibitor cocktail (Nakalai tesque). The lysate was cleared by centrifugation for 5 min at 20,000×g at 4°C and the supernatant was transferred to a new tube. 20 mL of NeutrAvidin beads (Thermo Fisher Scientific) was added into the cell lysate containing 300 mg of proteins and the mixture was rotating at 4°C for 2 h. After washing the beads with lysis buffer, the beads was suspended in 50 mL of SDS-PAGE sample buffer and subjected to Western blotting with 6D11 anti-PrP Ab.

### Transfection

Cells were transiently transfected with pcDNA-Sortilin and pcDNA-SortilinΔC at the final concentration of 1.6 μg/ml using lipofectamin 2000 (Invitrogen). The expression of sortilin and PrP^C^ was knocked down using Stealth RNAi siRNAs (Invitrogen): Sortilin siRNA #1, 5’-ccaagucaaauucugucccuauuau-3’; Sortilin siRNA #2, 5’-gagaacucuggaaaggugauacuaa-3’; PrP siRNA #1, 5’-gggacaaccucauggugguaguugg-3’; PrP siRNA #2, 5’-ccaguggaucaguacagcaaccaga-3’. Stealth RNAi Negative Control Duplex was purchased from Invitrogen. Each siRNA was transfected into cells at the final concentration of 10 nM using lipofectamin RNAiMax (Invitrogen).

### RT-PCR

Total RNA was extracted using an RNeasy Mini Kit (QIAGEN, Hilden, Germany) and first-strand cDNA was synthesized using SuperScript III First-Strand Synthesis System for RT-PCR (Invitrogen). The synthesized cDNAs were amplified with the following primer sets: 5’-aagcaggactcccgcccacag-3’ and 5’-ttccaggaggtcctcatctga-3’ for sortilin cDNA, 5’-tacagcaaccagaacaac-3’ and 5’-tcatcccacgatcaggaagat-3’ for PrP cDNA, and 5’-cctgccaagtatgatgacatc-3’ and 5’-gctgtagccgtattcattgtc-3’ for glyceraldehyde-3-phosphate dehydrogenase cDNA.

### Fractionation of membrane microdomains

Cells grown to ~80% confluency in a 35 mm tissue culture dish were suspended in 250 μL of MBS buffer [25 mM MES-NaOH (pH 6.5), 0.15 M NaCl] containing 1% (w/v) Triton X-100, and homogenized by being passed through a 21G-needle 15 times. After centrifugation at 500×g for 5 min at 4°C, 220 μL of the supernatant was transferred to a new tube and mixed with 220 μL of MBS buffer containing 80% (w/v) sucrose to make 40% (w/v) sucrose. 200 μL of the sample was placed at the bottom of a discontinuous sucrose gradient consisting of 600 μL of 30% (w/v) sucrose and 200 μL of 5% (w/v) sucrose. The sample was centrifuged at 140,000×g for 24 h at 4°C in an S55S rotor (Hitachi Koki, Tokyo, Japan). Ten fractions (100 μL/fraction) were collected from the top.

### Prion infection

Brains were removed from terminally ill wild-type C57BL/6 mice infected with RML prions. A single brain was homogenized (10%, w/v) in PBS using a multi-beads shocker (Yasui Kikai, Osaka, Japan) and then diluted to 1% with PBS. Two 1% (w/v) brain homogenates were mixed to prepare the homogenate of 2 pooled brains and the resulting homogenate was intracerebrally inoculated into a 4–5 week-old mouse with its 20 μL aliquot. The signs for disease-related symptoms were evaluated as previously described [37].

For infection of cells, cells were seeded at a density of 2×10^5^ cells/well in a 6-well tissue culture plate. At 4 h after cell seeding, the clarified RML-infected brain homogenate [34] containing 50 μg proteins was added to the well and cells were subsequently passaged every 3 days.

### Immunohistochemistry

Paraffin-embedded samples were sectioned, deparaffinized, and rehydrated. The samples were autoclaved in 1 mM HCl at 121°C for 5 min and subsequently washed with PBS. The samples were then digested with 50 μg/mL PK in PBS at 37°C for 30 min, treated with 3 M guanidine thiocyanate for 10 min, and washed with PBS. After blocking with 5% FBS in PBS for 1 h, the sampled were incubated with 6D11 anti-PrP Ab for 2 h, washed with PBS, and treated with ImmPRESS REAGENT Anti-Mouse IgG (Vector Laboratories, U.S.A) for 30 min. After washing with PBS, the samples were incubated with ImmPACT DAB (Vector Laboratories) for 180 sec for staining.

### Western blotting

Western blotting was performed as reported previously [26]. To evaluate protein expression, signals were densitometrically measured using LAS-4000 mini (Fujifilm Co., Tokyo, Japan). The measured intensity of target proteins was normalized against the signal intensity of β-actin used as an internal control protein.

### NGF stimulation

Cells were removed from a tissue culture dish by pipetting and transferred to a new tube. After washing with DMEM medium, cells were collected by centrifugation at 500×g for 2 min at 4°C, suspended in 1 mL of DMEM medium containing 2 nM NGF (Thermo Fisher Scientific), transferred into a well of 12 well plate, and incubated for 10 min at 37°C in a 5% CO_2_ incubator. The cells were collected by centrifugation at 500×g for 5 min at 4°C and washed with ice-cold PBS. The pellet was lysed in 200 μL of buffer B containing protease inhibitor cocktail (Nakalai tesque). The lysate was cleared by centrifugation at 12,000×g for 2 min at 4°C and the supernatant was subjected to Western blotting.

### Isolation of exosomes

Cells were cultured in 2 mL of DMEM medium containing 10% exosome-depleted FBS (System Bioscience, CA,USA) in a 6 well tissue culture plate for 72 h and the culture medium was collected. The culture medium was centrifuged at 2,000×g for 10 min at 4°C. The supernatant was passed through 0.22 μm pore filter membrane and the flow-through was centrifuged at 10,000×g for 30 min at 4°C. Exosomes in the supernatant were collected by ultracentrifugation at 100,000×g for 1 h at 4°C and washed with PBS. The exosomes were dissolved in 100 μL of Laemmli’s sample buffer and subjected to Western blotting.

### Detection of the C1 fragment

Cell lysate containing 20 μg proteins was incubated in 20 μL of (1×) glycoprotein denaturing buffer (0.5% SDS, 40mM DTT) at 99°C for 10 min. Thereafter, 3μl of 10% NP-40, 3 μL of (10×) Glycobuffer, 3.5 μl of distilled water and 0.5 μL of PNGase (500 units/μl) (New England BioLabs, MA, USA) were added. After 60 min incubation at 37°C, samples were mixed with 10 μL of (4×) Laemmli’s sample buffer and subjected to Western blotting.

### Statistical analysis

Survival and incubation times are analyzed using the log-rank test. Other data were analyzed using the one-way ANOVA.

## Supporting information

S1 Table Incubation and survival times of Sort1^-/-^ and Sort1^+/+^ female mice intracerebrally inoculated with RML prion. (DOCX)

S2 Table Incubation and survival times of Sort1^-/-^ and Sort1^+/+^ male mice intracerebrally inoculated with RML prion. (DOCX)

S1 Fig PrP^C^ interacts with sortilin but not with other VPS10P receptors. Co-immunoprecipitation assay was carried out in N2aC24 cells with SAF61 anti-PrP Ab. The resulting immunoprecipitates were subjected to Western blotting with Abs against each protein. Arrows and arrowheads indicate non-specific signals of the degraded fragment of protein G or the light chain of Abs used in co-immunoprecipitation.
(TIF)

S2 Fig Sortilin interacts with PrP^C^ through residues 610–753. (A) Schematic diagrams of full-length (full) sortilin and various deletion mutants of sortilin, all of which are tagged with a mycHis motif. Sortilin is a single-pass transmembrane molecule consisting of a signal peptide (red), a propeptide (yellow), a VPS10P domain (green), and a transmembrane region (blue). Arabic numbers represent the codon numbers. (B) Immunoprecipitation assay of sortilin-KO ΔSort#1 cells expressing full-length (full) sortilin and various deletion mutants of sortilin and of PrP-KO ΔPrP#1 cell expressing full-length (full) sortilin using SAF61 anti-PrP Ab. Immunoprecipitates (IP) and the cell lysates (Lysate) were subjected to Western blotting for sortilin with anti-myc Ab and for PrP^C^ with 6D11 anti-PrP Ab. An arrow indicates light chains of the Ab used in this assay.
(TIF)

S3 Fig Interaction of PrP^C^ and sortilin. Orthogonal views of double immunofluorescence staining of PrP^C^ (green) and sortilin (red) in non-permeabilized or permeabilized N2aC24 cells, with SAF83 anti-PrP Ab and goat polyclonal anti-sortilin Abs.
(TIF)

S4 Fig PrP^C^ interacts with sortilin on the cell surface. (A) A simple description of the protocol used for detection of interaction of PrP^C^ with sortilin on the cell surface. (B) Western blotting for PrP^C^ and sortilin in the immunocomplexes of SAF61 anti-PrP Ab from N2aC24 and ΔPrP#1 cells. (C) Western blotting for sortilin expressing in N2aC24 and ΔPrP#1 cells.
(TIF)

S5 Fig PrP^C^ is increased in the brains of Sort1^-/-^ mice. (A) Western blotting of the brains of WT (Sort1^+/+^) and Sort1^-/-^ mice for PrP^C^ with 6D11 anti-PrP Ab. Sortilin was detected in Sort1^+/+^ brains but not in Sort1^-/-^ brains. (B) Quantification of PrP^C^ densities after normalization against β-actin intensities in (A). Data are means ± SD of 3 brains. *** p < 0.001.
(TIF)

S6 Fig Shading of PrP^C^ and excretion of PrP^C^ in exosomes are increased in sortilin-deficient cells. (A) Western blotting for deglycosylated PrP^C^ in N2aC24 cells transfected with control and sortilin siRNAs. Full-length deglycosylated PrP^C^ and the C1 fragment were detectable. Quantification of densities for full-length deglycosylated PrP^C^ and the C1 fragment in (A). Data are means ± SD of 3 independent samples. ** p < 0.01. (B) Western blotting of the cell lysates and exosomes from N2aC24 cells and sortilin-KO ΔSort#1 and #2 cells for PrP^C^ with 6D11 anti-PrP Ab. TSG101 and flotillin-1, but not GM130 and Bcl-2, were detectable in exosomes. (C) Quantification of PrP^C^ densities in (B). Data are means ± SD of 3 independent samples. ** p < 0.01, *** p < 0.001.
(TIF)

S7 Fig Localization of PrP^C^ in late endosomes, recycling endosomes, and early endosomes. Double immunofluorescence staining of PrP^C^ (green) with the late endosome marker Rab9 (red) (A), the recycling endosome marker Rab11 (red) (C), and the early endosome marker EAA1 (red) (E). Pearson’s correlation coefficient for co-localization of PrP^C^ and Rab9 (B), Rab11 (D) or EAA1 (F). Data are means ± SD of 6 cells. ** p < 0.01, *** p < 0.001.
(TIF)

S8 Fig Impaired trafficking of PrPΔ23–88 to lysosomes. (A) Western blotting of full-length wild-type PrP^C^ and PrPΔ23–88 in WT cells and Δ23–88 cells after 12 h-treatment with or without 20 mM NH_4_Cl. (B) Quantification of wild-type PrP^C^ and PrPΔ23–88 in (A) after normalization against β-actin. Signal intensities in each lane were evaluated against that in NH_4_Cl-untreated WT#1 cells. Data are means ± SD of 4 independent experiments. * p < 0.05, *** p < 0.001. (C) Double immunofluorescence staining for PrP^C^ and PrPΔ23–88 with the lysosome marker LAMP1 in WT and Δ23–88 cells after 12 h-treatment with or without 20 mM NH_4_Cl. (D) Pearson’s correlation coefficients for co-localization of PrP^C^ or PrPΔ23–88 and LAMP1 in WT#1 cells untreated (n = 149) or treated (n = 120) with NH_4_Cl, WT#2 cells treated (n = 121) or untreated (n = 130) with NH_4_Cl, Δ23–88#1 cells treated (n = 124) or untreated (n = 138) with NH_4_Cl, and Δ23–88#1 cells treated (n = 122) or untreated (n = 121) with NH_4_Cl. Data are means ± SD. *** p < 0.001. (E) Western blotting for sortilin in WT and Δ23–88 cells. (F) Quantification of sortilin in (E) after normalization against β-actin. Signal intensities in each lane were evaluated against that in WT#1 cells. Data are means ± SD of 4 independent experiments. (G) Co-immunoprecipitation assay for PrP^C^ or PrPΔ23–88 and sortilin using SAF61 anti-PrP Ab. Arrows and arrowheads indicate non-specific signals of the degraded fragment of protein G or the light chain of Abs used in co-immunoprecipitation.
(TIF)

S9 Fig Membrane microdomain distribution of PrPΔ23–88 in cells and PrP^C^ in the brains of Sort1^-/-^ mice. (A) PrP-KO N2aΔPrP cells expressing WT PrP^C^, designated WT#1 and #2 cells, and those expressing PrPΔ23–88, Δ23–88#1 and #2 cells, were subjected to discontinuous sucrose gradient centrifugation. Each fraction was analyzed by Western blotting with 6D11 anti-PrP Ab. (B) Quantification of PrP^C^ or PrPΔ23–88 in each fraction against the total PrP^C^ or PrPΔ23–88 in (A). The signal density in each lane was evaluated against the total signal density of all lanes. Data are means ± SD of 3 independent experiments. (C) Discontinuous sucrose gradient centrifugation of the brains from Sort1^-/-^ and WT mice. Each fraction was analyzed by Western blotting with 6D11 anti-PrP antibody. (D) Quantification of PrP^C^ in each fraction against the total PrP^C^ in (C). The signal density in each lane was evaluated against the total signal density of all lanes. Data are means ± SD of 3 brains.
(TIF)

S10 Fig Membrane microdomain distribution of PrP molecules in prion-infected cells. (A) Discontinuous sucrose gradient centrifugation of prion-infected N2aC24L1-3 cells. Each fraction was treated with or without PK and analyzed by Western blotting with 6D11 anti-PrP Ab. (B) Quantification of PrP in each fraction against the total PrP in (A). The signal density in each lane was evaluated against the total signal density of all lanes. Data are means ± SD of 3 independent experiments.
(TIF)

S11 Fig PrP^Sc^ degradation is delayed in sortilin-KO cells infected with prions. (A) PrP^Sc^ in RML-infected N2aC24 (N2aC24/RML) and ΔSort#1 (ΔSort/RML) cells 36, 48, and 60 h after transfection with control siRNA alone. (B) Quantification of PrP^Sc^ in (A) after normalization against β-actin. Signal density in cells at 48 and 60 h was compared with that at 36 h. Data are means ± SD of 3 independent experiments. n.s., not significant. (C) PrP^Sc^ in 22L prion-infected N2aC24 (N2aC24/22L) and ΔSort#1 (ΔSort/22L) cells 36, 48, and 60 h after transfection with control siRNA or PrP-specific siRNAs (#1 and 2). (D) Quantification of PrP^Sc^ in (C) after normalization against β-actin. Each signal intensity in PrP-knockdown cells was evaluated against that in control siRNA-transfected cells in each blot. Data are means ± SD of 3 independent experiments.
(TIF)

S12 Fig Sortilin expression is decreased in prion-infected cells. (A) Western blotting of VPS10P receptors in infected N2aC24L1-3 cells and uninfected N2aC24. (B) Quantification of the VPS10P receptors in (A) after normalization against β-actin. The signal density in infected cells was evaluated against that in uninfected cells. Data are means ± SD of 3 independent experiments. n.s., not significant. (C) RT-PCR for sortilin, PrP, and GAPDH in infected N2aC24L1-3 and uninfected N2aC24 cells. (D) Western blotting of sortilin in N2a and ScN2a cells. (E) Quantification of sortilin in (D) after normalization against β-actin. The signal density in ScN2a was evaluated against that in N2a cells. Data are means ± SD of 4 independent experiments. *** p < 0.001.
(TIF)

S13 Fig Lysosomal degradation of sortilin is enhanced in prion-infected cells. (A) Western blotting of sortilin in N2aC24 and N2aC24L1-3 cells 12 h after treatment with 20 mM NH_4_Cl and 10 μM MG132. (B) Quantification of sortilin in (A) after normalization against β-actin. The signal intensity of sortilin in NH_4_Cl- or MG132-treated cells was evaluated against that in untreated cells. Data are means ± SD of 3 independent experiments. n.s, not significant; ** p < 0.01, *** p < 0.001. (C) Western blotting for sortilin in N2aC24L1-3 cells 12 h after treatment with 20 mM NH_4_Cl or 10 nM Concanamycin A. (D) Quantification of sortilin in (C) after normalization against β-actin. The signal intensity of sortilin in NH_4_Cl- or Concanamycin A-treated cells was evaluated against that in untreated cells. Data are means ± SD of 3 independent experiments. n.s, not significant; *** p < 0.001.
(TIF)

S14 Fig Impaired activation of ERK1/2 in prion-infected cells. (A) Western blotting of uninfected N2aC24 and prion-infected N2aC24L1-3 cells for phosphorylated ERK1/2 and total ERK1 after treatment with or without NGF. (B) Quantification of phosphorylated ERK1/2 densities after normalization against β-actin densities in (A). Data are means ± SD of 3 independent experiments. *** p < 0.001. (C) Quantification of total ERK1 intensities in (A) after normalization against β-actin intensities. Data are means ± SD of 3 independent experiments.
(TIF)

S15 Fig Sortilin-KO male mice have accelerated prion disease with earlier accumulation of PrP^Sc^ in their brains. (A) Kaplan-Meier survival curves for Sort1^-/-^ (n = 21) and Sort1^+/+^ (n = 23) male mice inoculated with RML prions. *** p < 0.001. (B) Western blotting of PrP^Sc^ in the brains of Sort1^-/-^ and Sort1^+/+^ mice at 45, 60, and 90 dpi and at terminal stage. (C) Quantification of PrP^Sc^ in (B) after normalization against β-actin. Signal intensity in Sort1^-/-^ mice was evaluated against that in Sort1^+/+^ mice. Data are means ± SD of 4–6 independent brains. n.s., not significant; * p < 0.05. (D) Immunohistochemical staining of PrP^Sc^ in the brain hippocampus areas of Sort1^-/-^ and Sort1^+/+^ mice at 60 and 90 dpi and at terminal stage. Bar, 300 μm.
(TIF)
