# Supplementary material for: Prions amplify through degradation of the VPS10P sorting receptor sortilin
Source: PLoS Pathog. 2017 Jun 30;13(6):e1006470. doi: 10.1371/journal.ppat.1006470 (PMC5509376; doi:10.1371/journal.ppat.1006470)
Supplement: S1 Table — (DOCX) [file ppat.1006470.s001.docx]

S1 Table. Incubation and survival times of Sort1^-/-^ and Sort1^+/+^ female mice intracerebrally inoculated with RML prion.

Incubation period (days) Survival period (days) Diseased/inoculated

Sort1^-/-^ 150.9 ± 7.8 158.5 ± 6.3 24/24

Sort1^+/+^ 171.9 ± 6.0 179.7 ± 6.4 19/19
