# Supplementary material for: Prions amplify through degradation of the VPS10P sorting receptor sortilin
Source: PLoS Pathog. 2017 Jun 30;13(6):e1006470. doi: 10.1371/journal.ppat.1006470 (PMC5509376; doi:10.1371/journal.ppat.1006470)
Supplement: S2 Table — (DOCX) [file ppat.1006470.s002.docx]

S2 Table. Incubation and survival times of Sort1^-/-^ and Sort1^+/+^ male mice intracerebrally inoculated with RML prion.

Incubation period (days) Survival period (days) Diseased/inoculated

Sort1^-/-^ 163.0 ± 7.1 170.0 ± 6.0 21/21

Sort1^+/+^ 181.7 ± 6.4 188.0 ± 5.7 23/23
