# Supplementary figures and images for: Prions amplify through degradation of the VPS10P sorting receptor sortilin
Source: PLoS Pathog. 2017 Jun 30;13(6):e1006470. doi: 10.1371/journal.ppat.1006470 (PMC5509376; doi:10.1371/journal.ppat.1006470)

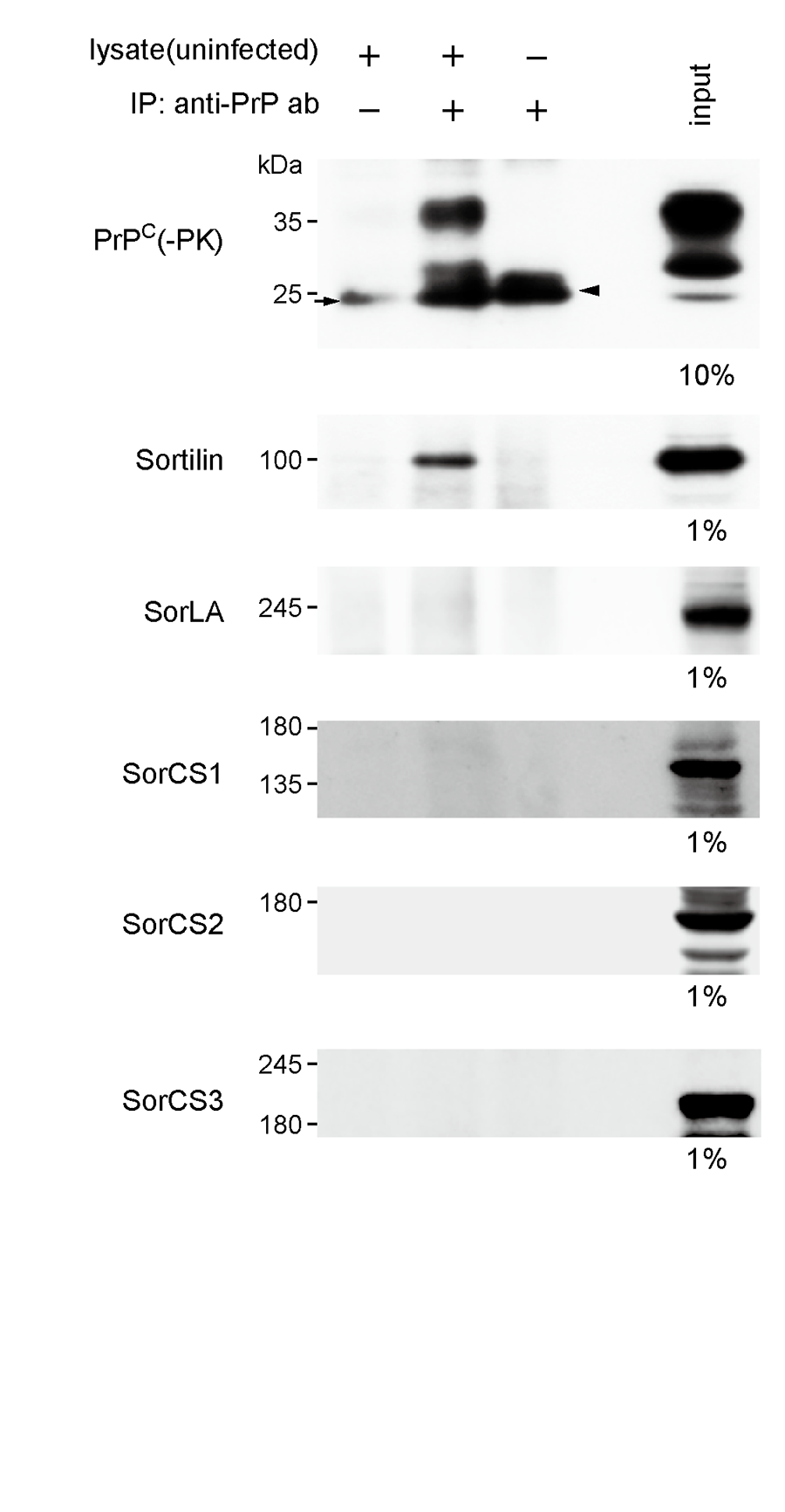

Supplement: S1 Fig — Co-immunoprecipitation assay was carried out in N2aC24 cells with SAF61 anti-PrP Ab. The resulting immunoprecipitates were subjected to Western blotting with Abs against each protein. Arrows and arrowheads indicate non-specific signals of the degraded fragment of protein G or the light chain of Abs used in co-immunoprecipitation. (TIF) [file ppat.1006470.s003.tif]

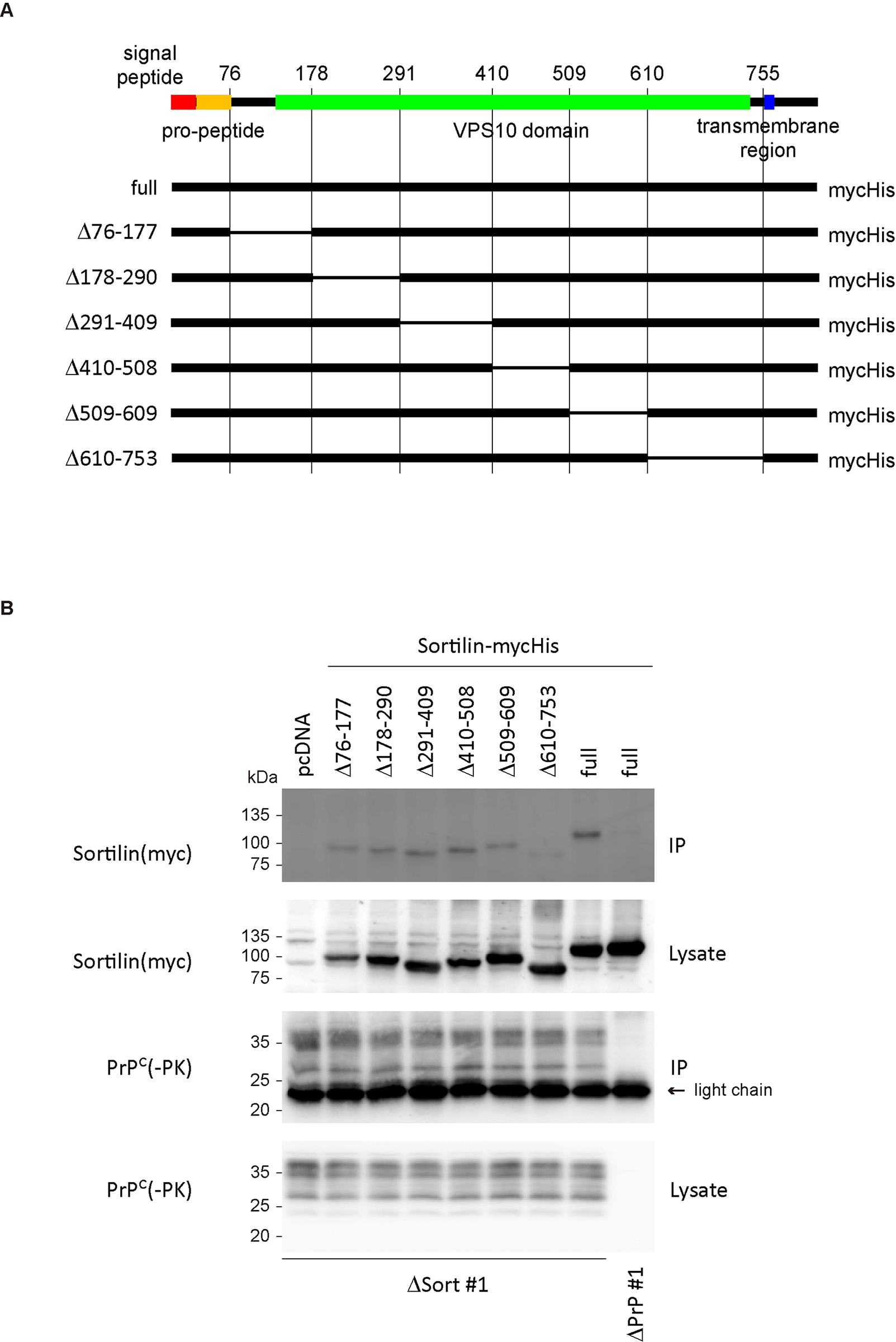

Supplement: S2 Fig — (A) Schematic diagrams of full-length (full) sortilin and various deletion mutants of sortilin, all of which are tagged with a mycHis motif. Sortilin is a single-pass transmembrane molecule consisting of a signal peptide (red), a propeptide (yellow), a VPS10P domain (green), and a transmembrane region (blue). Arabic numbers represent the codon numbers. (B) Immunoprecipitation assay of sortilin-KO ΔSort#1 cells expressing full-length (full) sortilin and various deletion mutants of sortilin and of PrP-KO ΔPrP#1 cell expressing full-length (full) sortilin using SAF61 anti-PrP Ab. Immunoprecipitates (IP) and the cell lysates (Lysate) were subjected to Western blotting for sortilin with anti-myc Ab and for PrPC with 6D11 anti-PrP Ab. An arrow indicates light chains of the Ab used in this assay. (TIF) [file ppat.1006470.s004.tif]

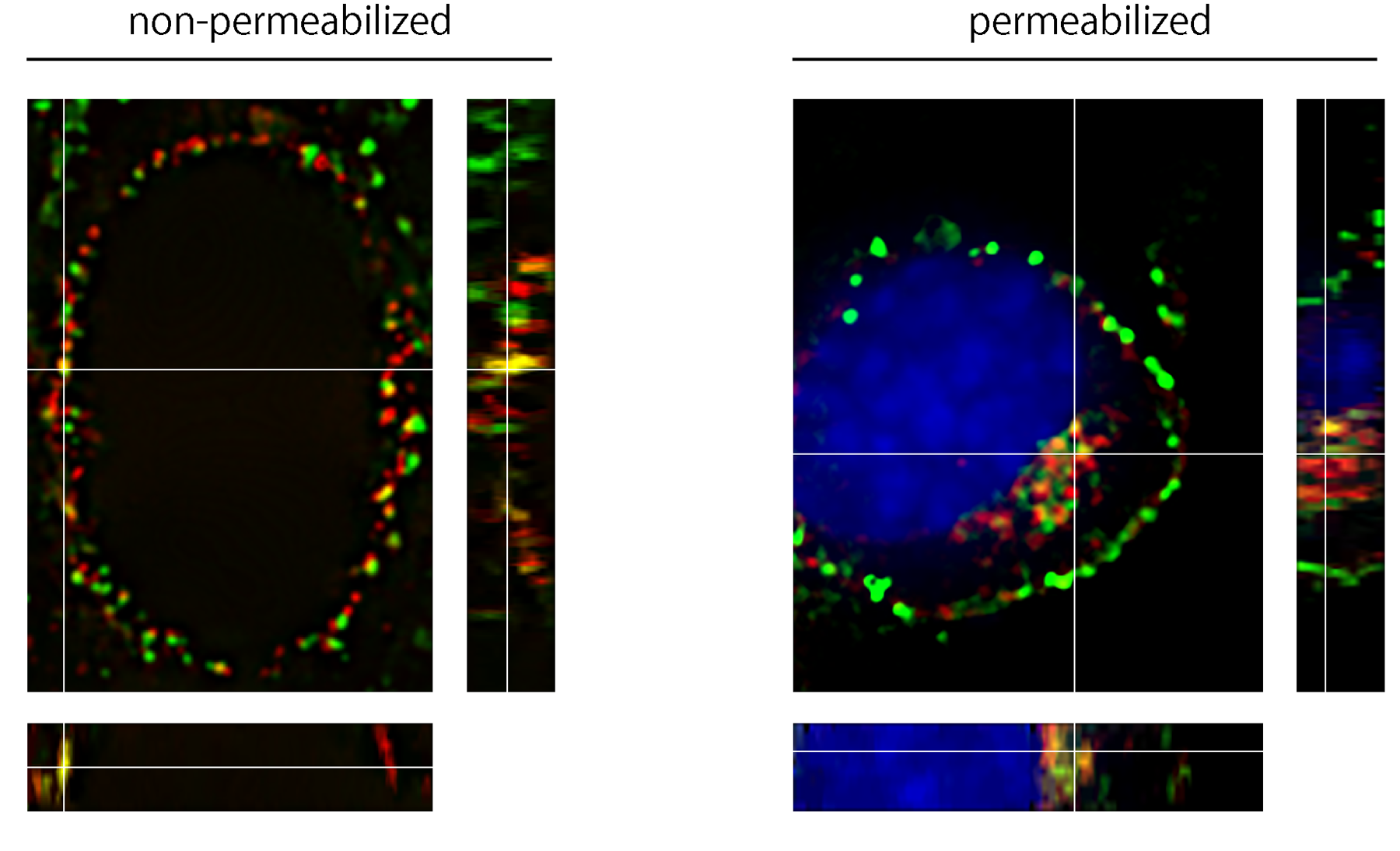

Supplement: S3 Fig — Orthogonal views of double immunofluorescence staining of PrPC (green) and sortilin (red) in non-permeabilized or permeabilized N2aC24 cells, with SAF83 anti-PrP Ab and goat polyclonal anti-sortilin Abs. (TIF) [file ppat.1006470.s005.tif]

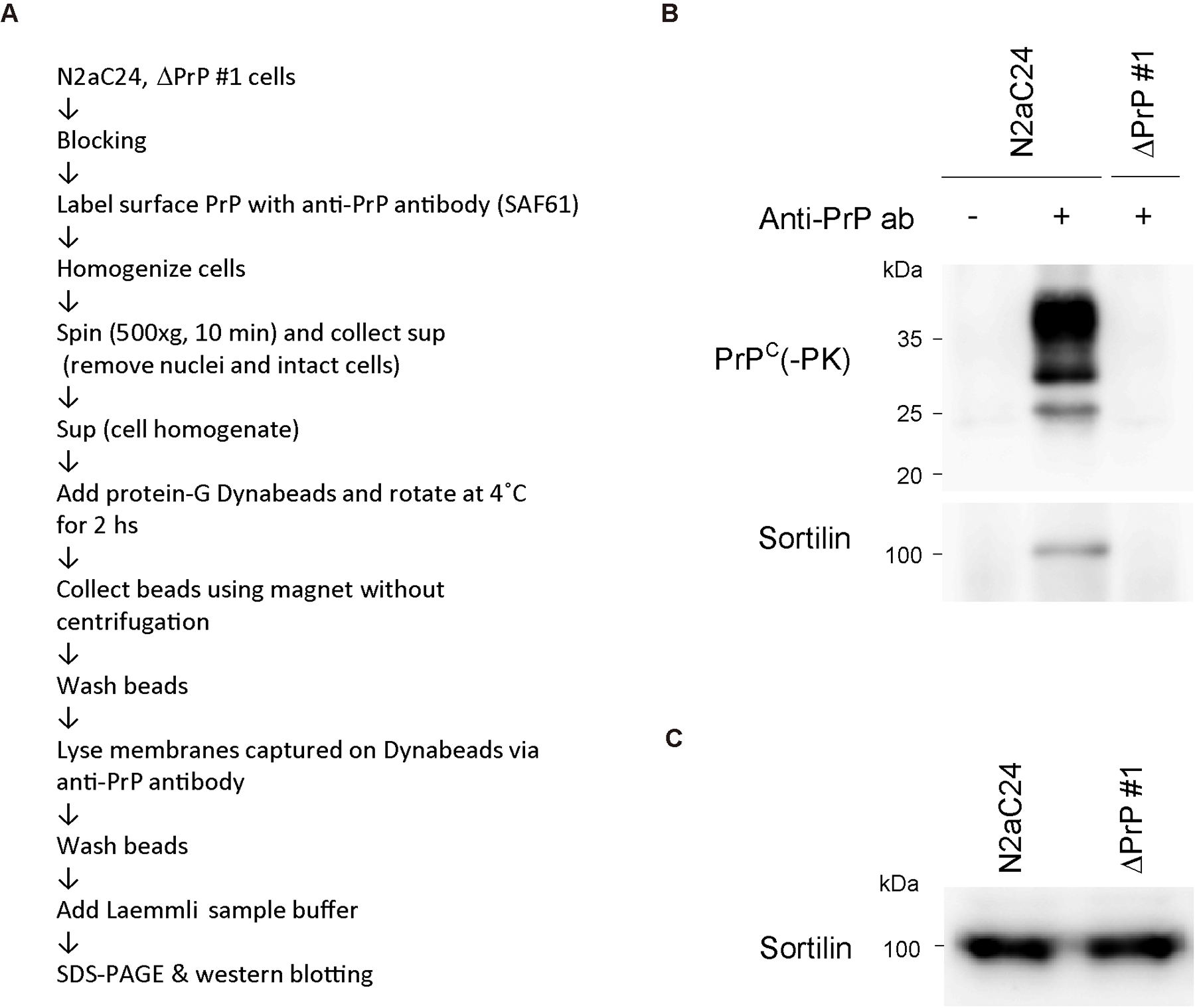

Supplement: S4 Fig — (A) A simple description of the protocol used for detection of interaction of PrPC with sortilin on the cell surface. (B) Western blotting for PrPC and sortilin in the immunocomplexes of SAF61 anti-PrP Ab from N2aC24 and ΔPrP#1 cells. (C) Western blotting for sortilin expressing in N2aC24 and ΔPrP#1 cells. (TIF) [file ppat.1006470.s006.tif]

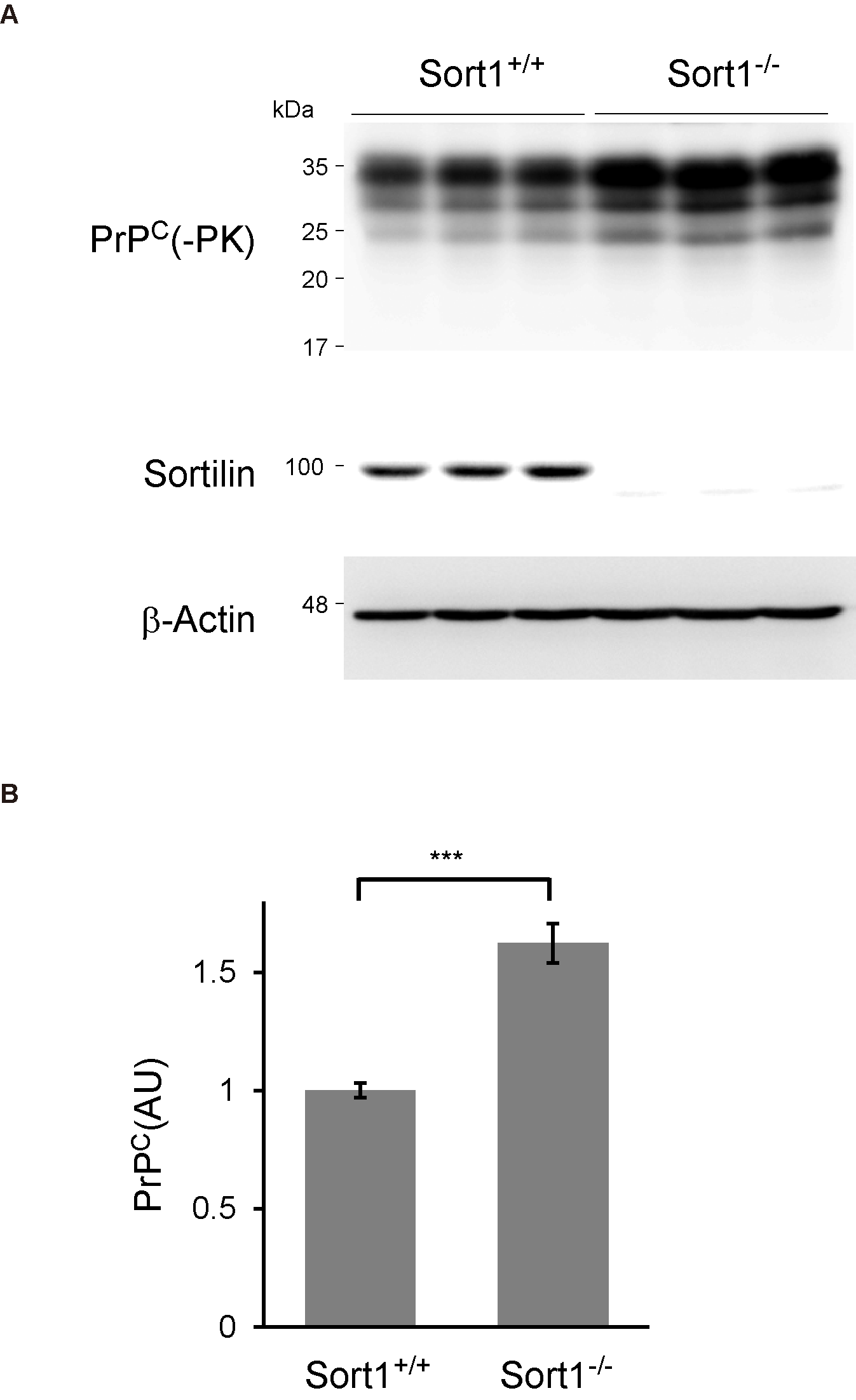

Supplement: S5 Fig — (A) Western blotting of the brains of WT (Sort1+/+) and Sort1-/- mice for PrPC with 6D11 anti-PrP Ab. Sortilin was detected in Sort1+/+ brains but not in Sort1-/- brains. (B) Quantification of PrPC densities after normalization against β-actin intensities in (A). Data are means ± SD of 3 brains. *** p < 0.001. (TIF) [file ppat.1006470.s007.tif]

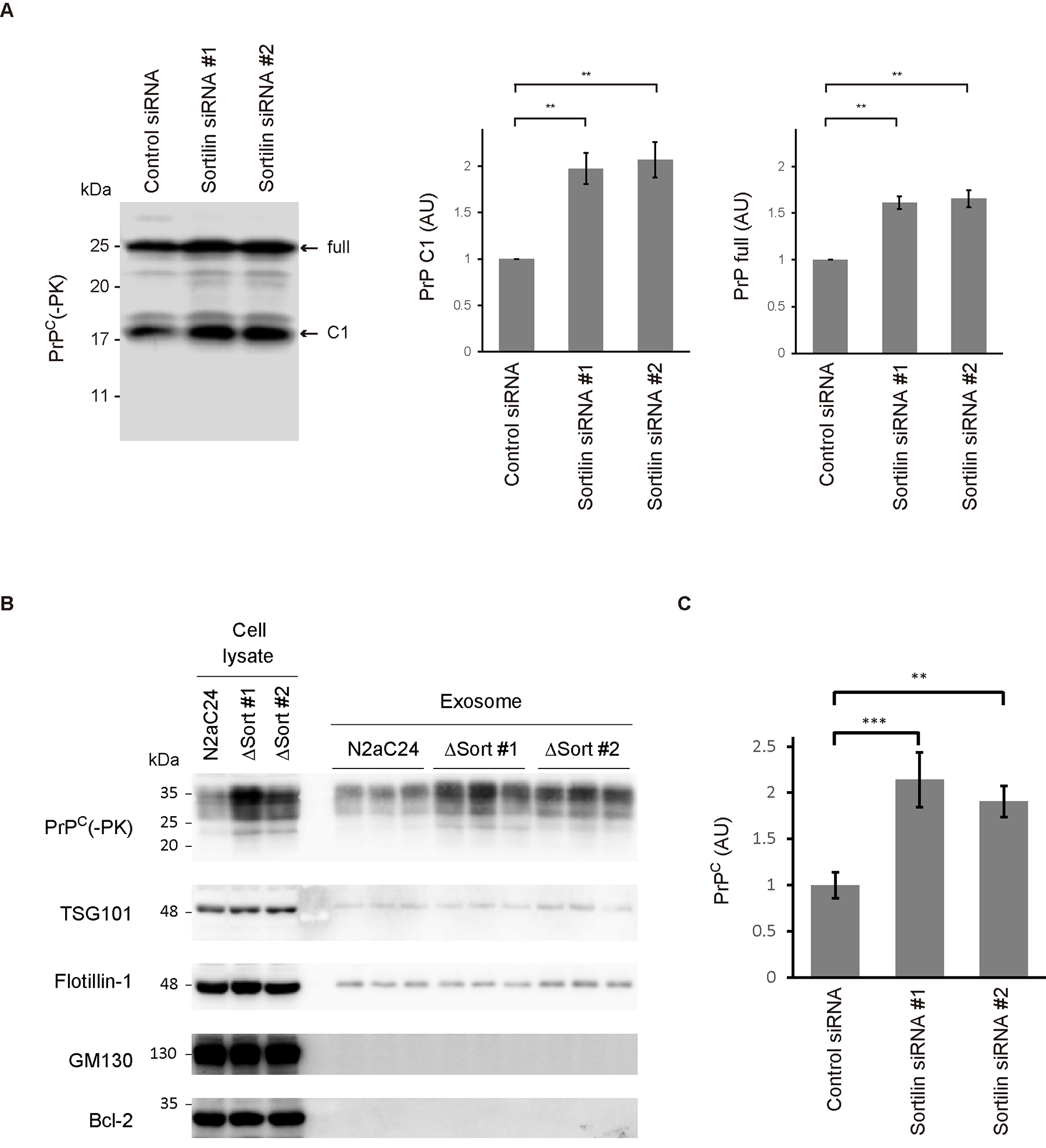

Supplement: S6 Fig — (A) Western blotting for deglycosylated PrPC in N2aC24 cells transfected with control and sortilin siRNAs. Full-length deglycosylated PrPC and the C1 fragment were detectable. Quantification of densities for full-length deglycosylated PrPC and the C1 fragment in (A). Data are means ± SD of 3 independent samples. ** p < 0.01. (B) Western blotting of the cell lysates and exosomes from N2aC24 cells and sortilin-KO ΔSort#1 and #2 cells for PrPC with 6D11 anti-PrP Ab. TSG101 and flotillin-1, but not GM130 and Bcl-2, were detectable in exosomes. (C) Quantification of PrPC densities in (B). Data are means ± SD of 3 independent samples. ** p < 0.01, *** p < 0.001. (TIF) [file ppat.1006470.s008.tif]

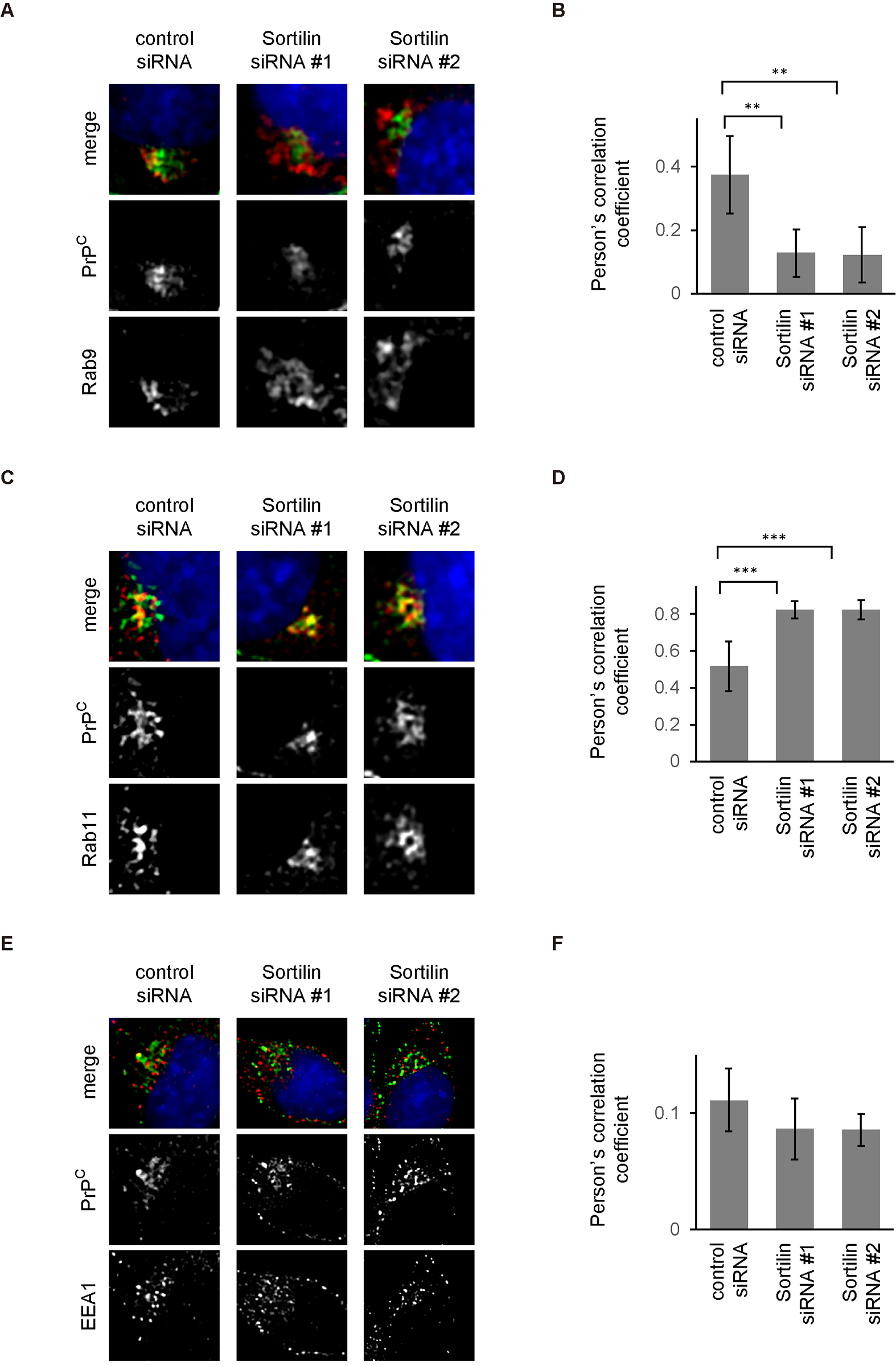

Supplement: S7 Fig — Double immunofluorescence staining of PrPC (green) with the late endosome marker Rab9 (red) (A), the recycling endosome marker Rab11 (red) (C), and the early endosome marker EAA1 (red) (E). Pearson’s correlation coefficient for co-localization of PrPC and Rab9 (B), Rab11 (D) or EAA1 (F). Data are means ± SD of 6 cells. ** p < 0.01, *** p < 0.001. (TIF) [file ppat.1006470.s009.tif]

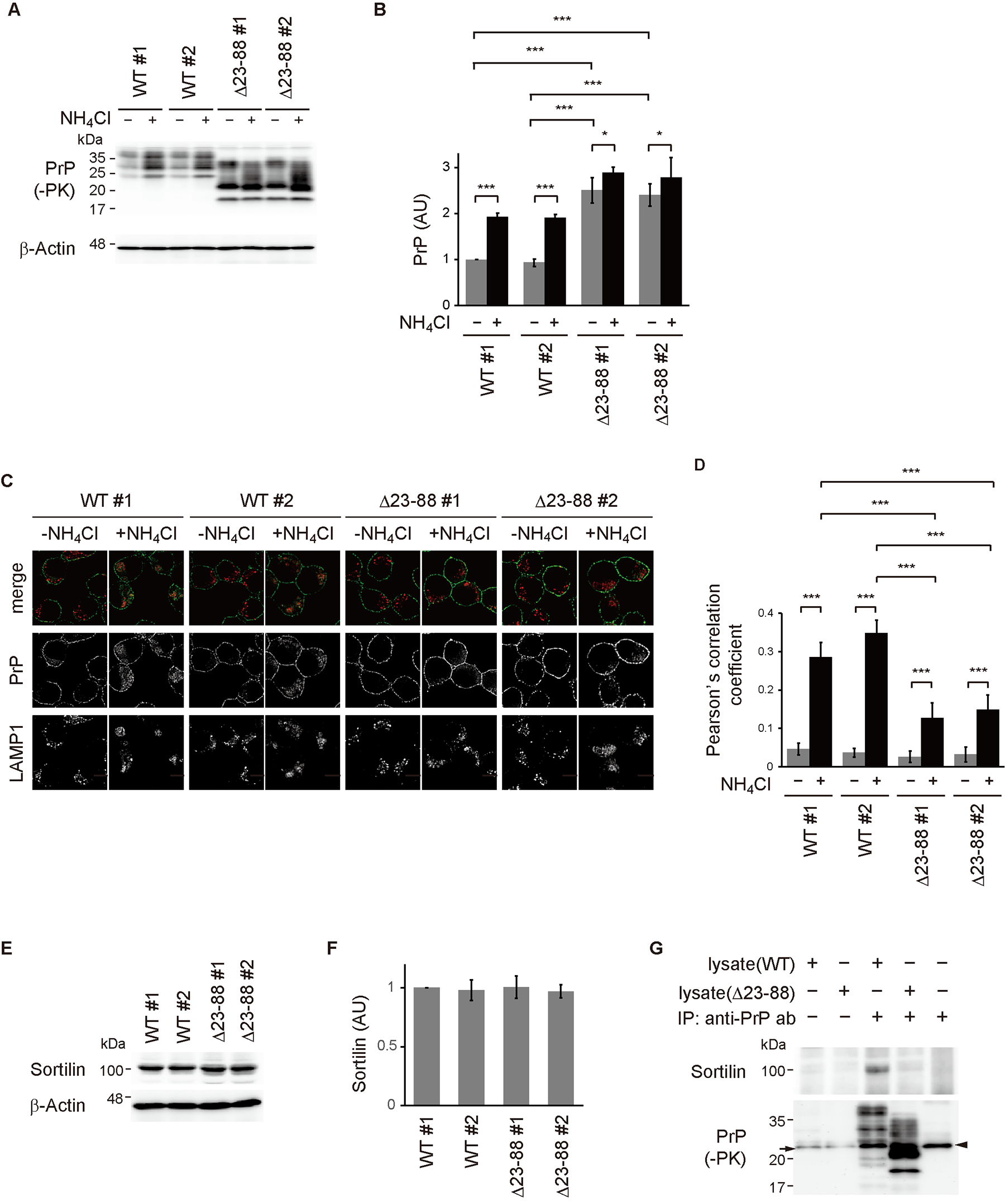

Supplement: S8 Fig — (A) Western blotting of full-length wild-type PrPC and PrPΔ23–88 in WT cells and Δ23–88 cells after 12 h-treatment with or without 20 mM NH4Cl. (B) Quantification of wild-type PrPC and PrPΔ23–88 in (A) after normalization against β-actin. Signal intensities in each lane were evaluated against that in NH4Cl-untreated WT#1 cells. Data are means ± SD of 4 independent experiments. * p < 0.05, *** p < 0.001. (C) Double immunofluorescence staining for PrPC and PrPΔ23–88 with the lysosome marker LAMP1 in WT and Δ23–88 cells after 12 h-treatment with or without 20 mM NH4Cl. (D) Pearson’s correlation coefficients for co-localization of PrPC or PrPΔ23–88 and LAMP1 in WT#1 cells untreated (n = 149) or treated (n = 120) with NH4Cl, WT#2 cells treated (n = 121) or untreated (n = 130) with NH4Cl, Δ23–88#1 cells treated (n = 124) or untreated (n = 138) with NH4Cl, and Δ23–88#1 cells treated (n = 122) or untreated (n = 121) with NH4Cl. Data are means ± SD. *** p < 0.001. (E) Western blotting for sortilin in WT and Δ23–88 cells. (F) Quantification of sortilin in (E) after normalization against β-actin. Signal intensities in each lane were evaluated against that in WT#1 cells. Data are means ± SD of 4 independent experiments. (G) Co-immunoprecipitation assay for PrPC or PrPΔ23–88 and sortilin using SAF61 anti-PrP Ab. Arrows and arrowheads indicate non-specific signals of the degraded fragment of protein G or the light chain of Abs used in co-immunoprecipitation. (TIF) [file ppat.1006470.s010.tif]

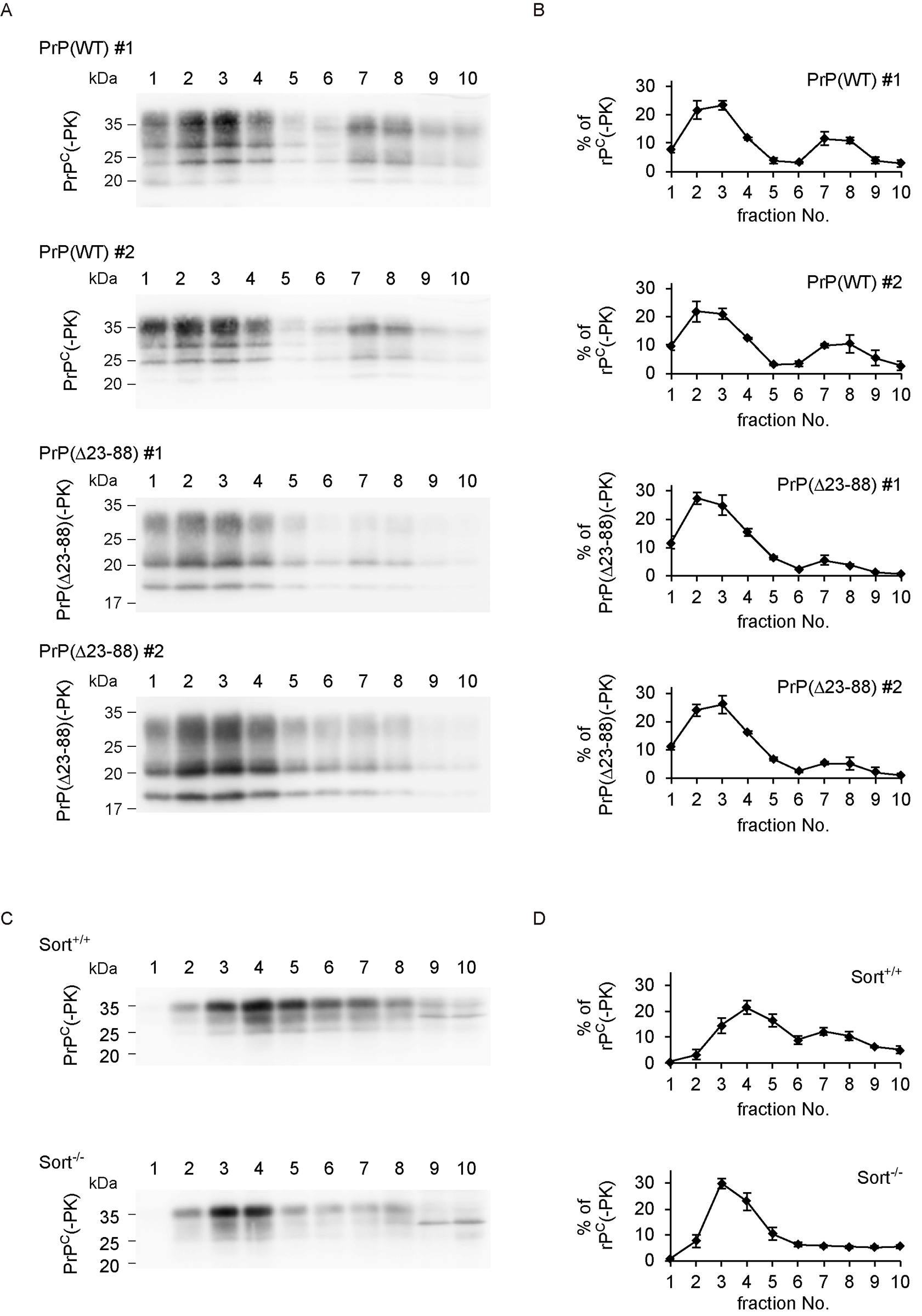

Supplement: S9 Fig — (A) PrP-KO N2aΔPrP cells expressing WT PrPC, designated WT#1 and #2 cells, and those expressing PrPΔ23–88, Δ23–88#1 and #2 cells, were subjected to discontinuous sucrose gradient centrifugation. Each fraction was analyzed by Western blotting with 6D11 anti-PrP Ab. (B) Quantification of PrPC or PrPΔ23–88 in each fraction against the total PrPC or PrPΔ23–88 in (A). The signal density in each lane was evaluated against the total signal density of all lanes. Data are means ± SD of 3 independent experiments. (C) Discontinuous sucrose gradient centrifugation of the brains from Sort1-/- and WT mice. Each fraction was analyzed by Western blotting with 6D11 anti-PrP antibody. (D) Quantification of PrPC in each fraction against the total PrPC in (C). The signal density in each lane was evaluated against the total signal density of all lanes. Data are means ± SD of 3 brains. (TIF) [file ppat.1006470.s011.tif]

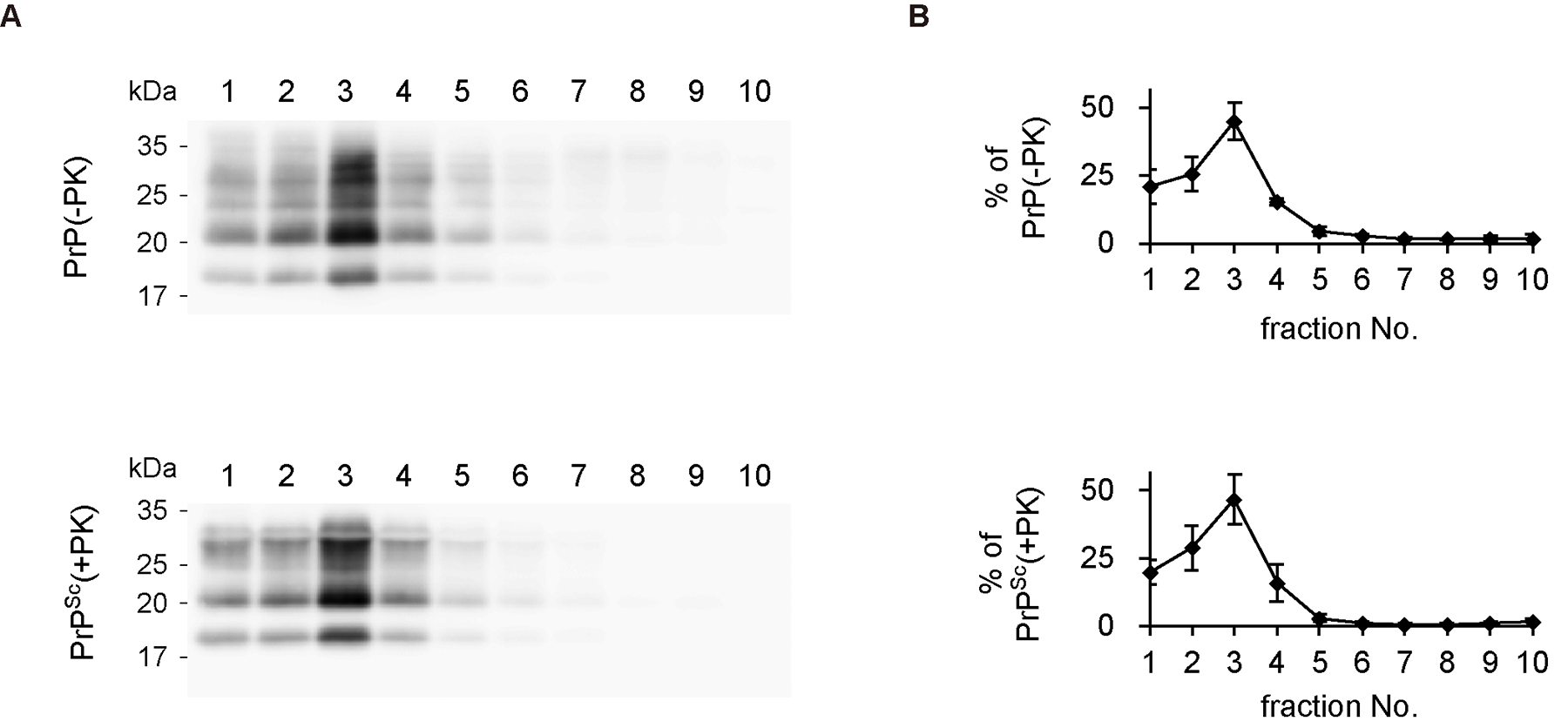

Supplement: S10 Fig — (A) Discontinuous sucrose gradient centrifugation of prion-infected N2aC24L1-3 cells. Each fraction was treated with or without PK and analyzed by Western blotting with 6D11 anti-PrP Ab. (B) Quantification of PrP in each fraction against the total PrP in (A). The signal density in each lane was evaluated against the total signal density of all lanes. Data are means ± SD of 3 independent experiments. (TIF) [file ppat.1006470.s012.tif]

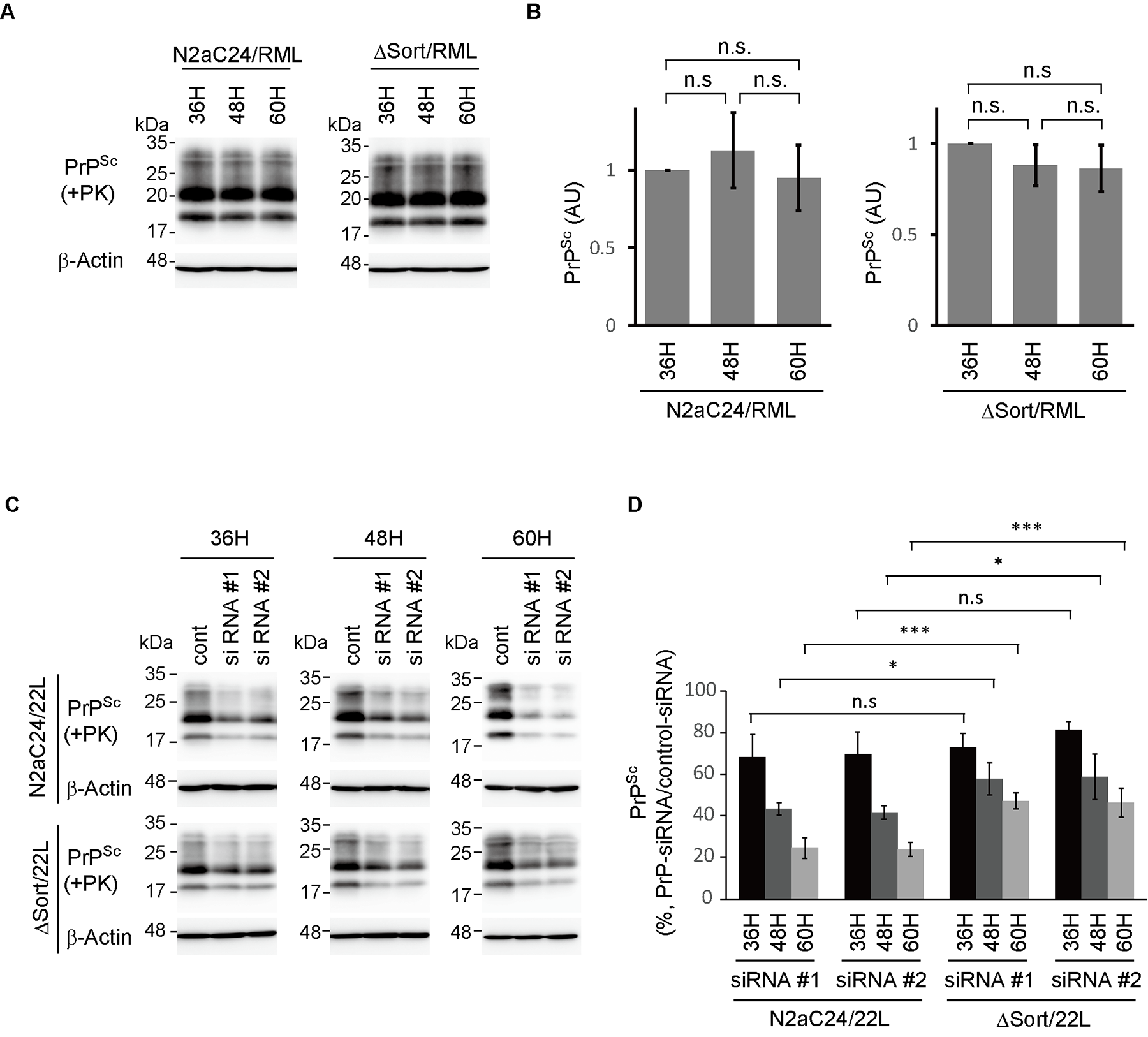

Supplement: S11 Fig — (A) PrPSc in RML-infected N2aC24 (N2aC24/RML) and ΔSort#1 (ΔSort/RML) cells 36, 48, and 60 h after transfection with control siRNA alone. (B) Quantification of PrPSc in (A) after normalization against β-actin. Signal density in cells at 48 and 60 h was compared with that at 36 h. Data are means ± SD of 3 independent experiments. n.s., not significant. (C) PrPSc in 22L prion-infected N2aC24 (N2aC24/22L) and ΔSort#1 (ΔSort/22L) cells 36, 48, and 60 h after transfection with control siRNA or PrP-specific siRNAs (#1 and 2). (D) Quantification of PrPSc in (C) after normalization against β-actin. Each signal intensity in PrP-knockdown cells was evaluated against that in control siRNA-transfected cells in each blot. Data are means ± SD of 3 independent experiments. (TIF) [file ppat.1006470.s013.tif]

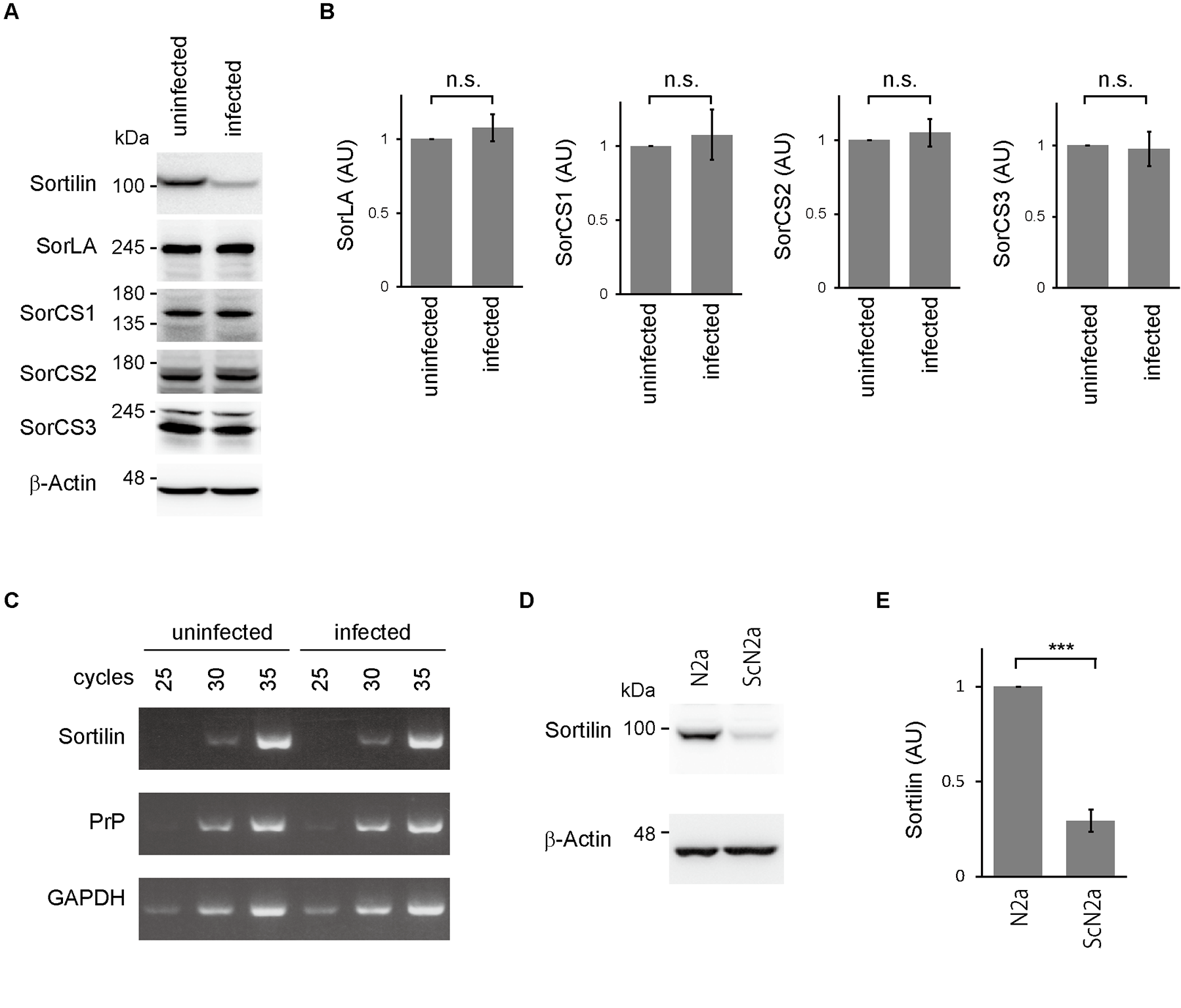

Supplement: S12 Fig — (A) Western blotting of VPS10P receptors in infected N2aC24L1-3 cells and uninfected N2aC24. (B) Quantification of the VPS10P receptors in (A) after normalization against β-actin. The signal density in infected cells was evaluated against that in uninfected cells. Data are means ± SD of 3 independent experiments. n.s., not significant. (C) RT-PCR for sortilin, PrP, and GAPDH in infected N2aC24L1-3 and uninfected N2aC24 cells. (D) Western blotting of sortilin in N2a and ScN2a cells. (E) Quantification of sortilin in (D) after normalization against β-actin. The signal density in ScN2a was evaluated against that in N2a cells. Data are means ± SD of 4 independent experiments. *** p < 0.001. (TIF) [file ppat.1006470.s014.tif]

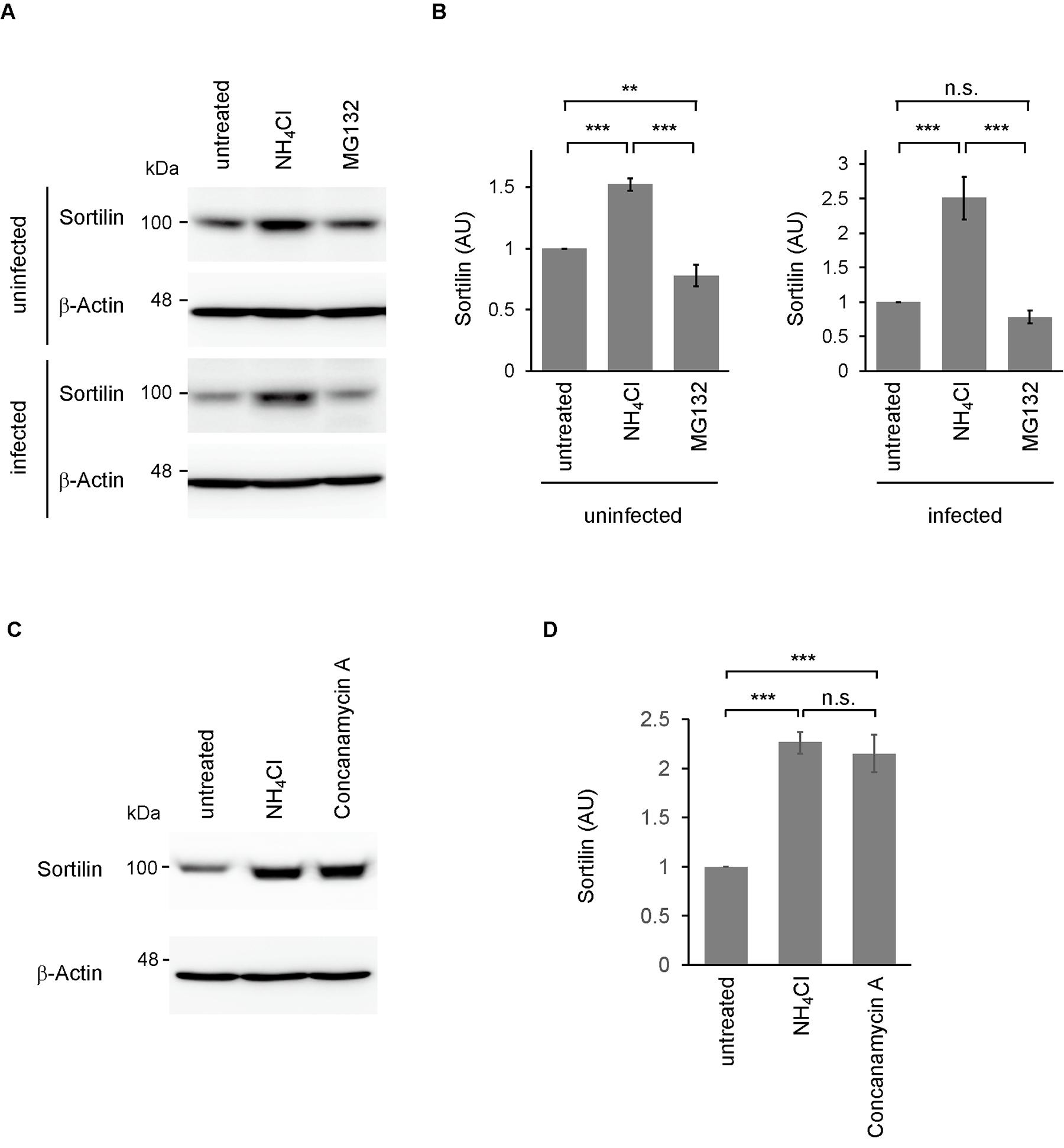

Supplement: S13 Fig — (A) Western blotting of sortilin in N2aC24 and N2aC24L1-3 cells 12 h after treatment with 20 mM NH4Cl and 10 μM MG132. (B) Quantification of sortilin in (A) after normalization against β-actin. The signal intensity of sortilin in NH4Cl- or MG132-treated cells was evaluated against that in untreated cells. Data are means ± SD of 3 independent experiments. n.s, not significant; ** p < 0.01, *** p < 0.001. (C) Western blotting for sortilin in N2aC24L1-3 cells 12 h after treatment with 20 mM NH4Cl or 10 nM Concanamycin A. (D) Quantification of sortilin in (C) after normalization against β-actin. The signal intensity of sortilin in NH4Cl- or Concanamycin A-treated cells was evaluated against that in untreated cells. Data are means ± SD of 3 independent experiments. n.s, not significant; *** p < 0.001. (TIF) [file ppat.1006470.s015.tif]

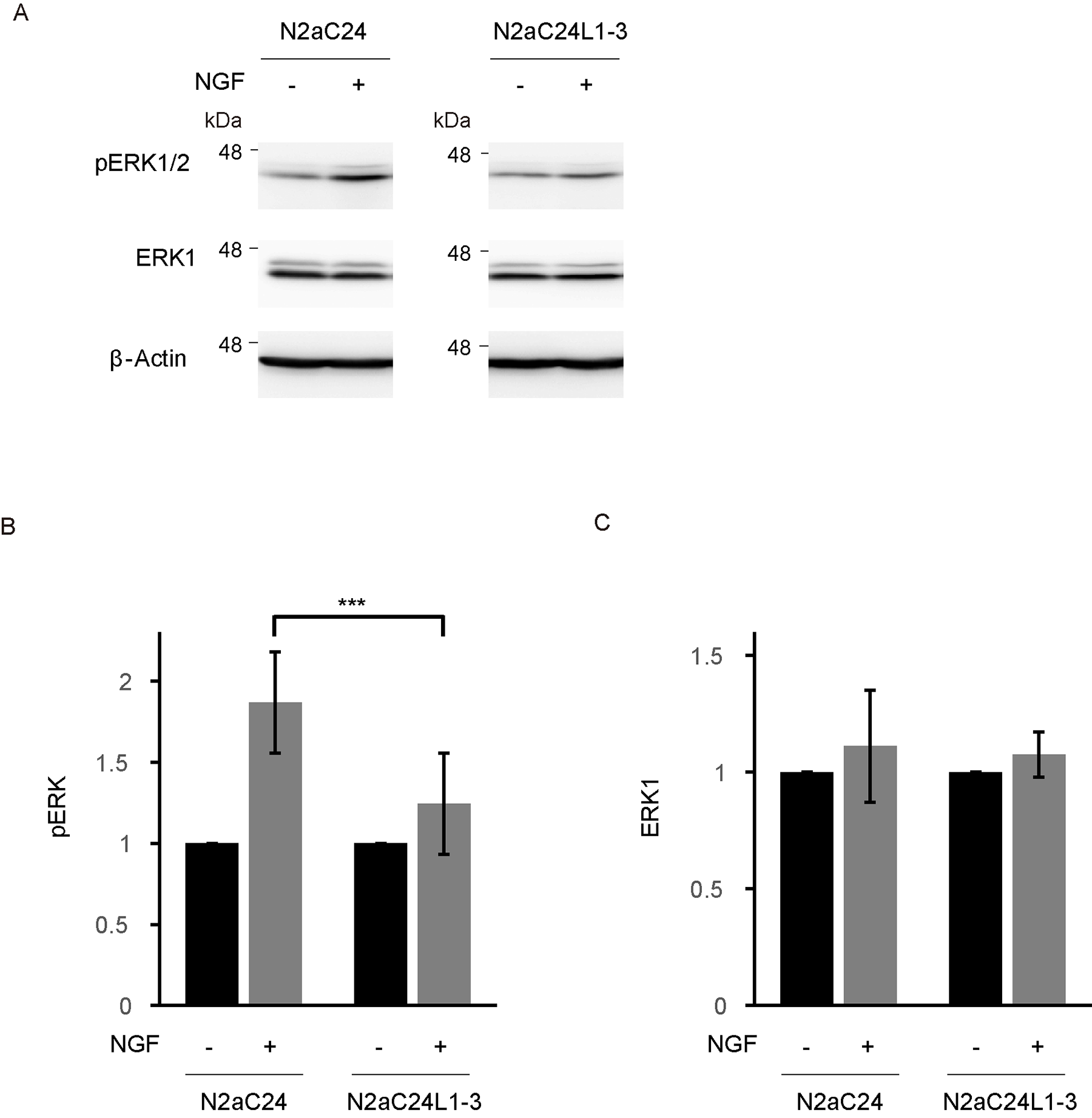

Supplement: S14 Fig — (A) Western blotting of uninfected N2aC24 and prion-infected N2aC24L1-3 cells for phosphorylated ERK1/2 and total ERK1 after treatment with or without NGF. (B) Quantification of phosphorylated ERK1/2 densities after normalization against β-actin densities in (A). Data are means ± SD of 3 independent experiments. *** p < 0.001. (C) Quantification of total ERK1 intensities in (A) after normalization against β-actin intensities. Data are means ± SD of 3 independent experiments. (TIF) [file ppat.1006470.s016.tif]

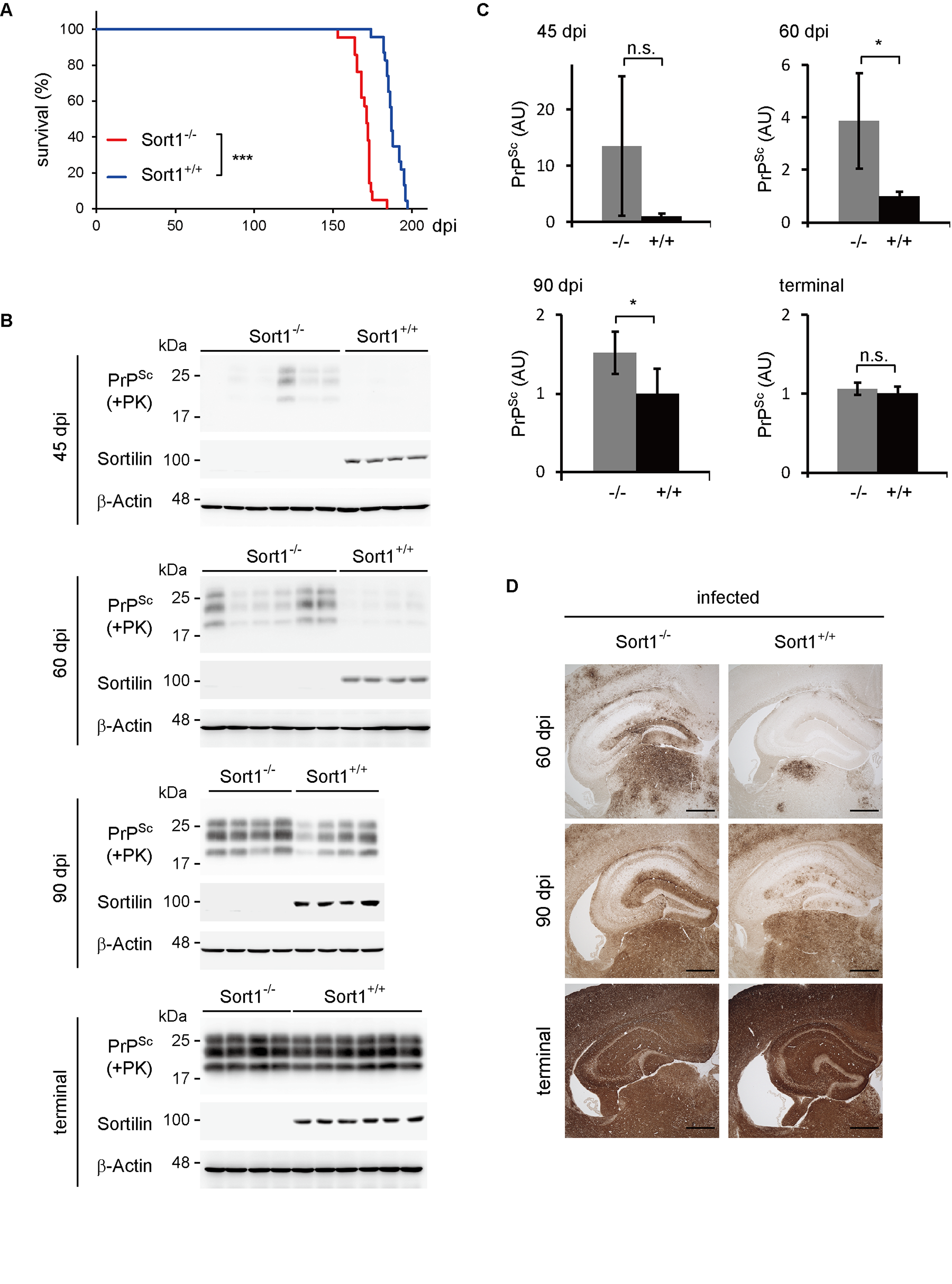

Supplement: S15 Fig — (A) Kaplan-Meier survival curves for Sort1-/- (n = 21) and Sort1+/+ (n = 23) male mice inoculated with RML prions. *** p < 0.001. (B) Western blotting of PrPSc in the brains of Sort1-/- and Sort1+/+ mice at 45, 60, and 90 dpi and at terminal stage. (C) Quantification of PrPSc in (B) after normalization against β-actin. Signal intensity in Sort1-/- mice was evaluated against that in Sort1+/+ mice. Data are means ± SD of 4–6 independent brains. n.s., not significant; * p < 0.05. (D) Immunohistochemical staining of PrPSc in the brain hippocampus areas of Sort1-/- and Sort1+/+ mice at 60 and 90 dpi and at terminal stage. Bar, 300 μm. (TIF) [file ppat.1006470.s017.tif]
